# HECTD1 Directs HIF‐1α to Facilitate Intestinal Inflammation and Carcinogenesis via Promoting Th17 Differentiation and Glycolysis

**DOI:** 10.1002/advs.78339

**Published:** 2026-10-18

**Authors:** Shuiyu Xu, Qiaoqiao Han, Feixiang Wang, Yuerong Zhang, Haoran Feng, Kun Liu, Fang Xiu Luo, Fei Yuan, Xuesong Zhao, Xiaoyan Yu, Haiyan Huang, Qiang Zou, Ren Zhao

**Affiliations:** ^1^ Department of General Surgery Ruijin Hospital Shanghai Jiao Tong University School of Medicine Shanghai China; ^2^ Shanghai Institute of Immunology Department of Immunology and Microbiology State Key Laboratory of Systems Medicine for Cancer Shanghai Jiao Tong University School of Medicine Shanghai China; ^3^ Department of Laboratory Medicine Institute of Laboratory Medicine School of Medicine Sichuan Provincial People's Hospital University of Electronic Science and Technology of China Chengdu China; ^4^ Department of Pathology Ruijin Hospital Shanghai Jiao Tong University School of Medicine Shanghai China; ^5^ Department of Radiology Ruijin Hospital Shanghai Jiao Tong University School of Medicine Shanghai China

**Keywords:** colitis‐associated cancer, glycolysis, HECTD1, inflammatory bowel disease, Th17 cells

## Abstract

Targeting glucose metabolism in Th17 cells may be beneficial for treating inflammatory bowel disease (IBD) and colitis‐associated cancer (CAC), however, the molecular mechanisms orchestrating Th17 cell metabolism remain unclear. Here, we revealed that HECTD1 regulates IGF2BP3‐dependent HIF‐1α translation to promote Th17 cell glycolysis and differentiation and that HECTD1 expression correlates with IBD and CAC pathogenesis. T‐cell‐specific deletion of HECTD1 mitigated the severity of colitis and CAC in murine models. RNA‐seq revealed enrichment of Th17 differentiation, glycolysis/gluconeogenesis, and HIF‐1 signaling pathways among differentially expressed genes between HECTD1‐sufficient and HECTD1‐deficient CD4^+^ T cells. Consistently, loss of HECTD1 suppressed Th17 cell differentiation both in vitro and in vivo, and suppressed glycolytic metabolism and HIF‐1α activity, revealing the mechanism underlying its protective effects against IBD and CAC. Mechanistically, HECTD1 potentiates m^6^A‐based HIF‐1α translation by mediating IGF2BP3 K63‐linked ubiquitination to promote the binding of IGF2BP3 with the translation initiation machinery of *Hif1a* mRNA. Reciprocally, HIF‐1α acts as a key transcription factor that promotes *Hectd1* gene expression, establishing a distinct positive feedback loop. Our findings highlight a self‐reinforcing feedback mechanism in which HECTD1 and HIF‐1α engage in mutual regulation to dictate Th17 cell fate and metabolic reprogramming, providing novel potential IBD and CAC treatments targeting HECTD1.

## Graphical abstract

HECTD1 emerges as an upstream amplifier of Th17 metabolic programming by promoting K63‐linked ubiquitination of IGF2BP3 and enhancing HIF‐1α translation. HIF‐1α reciprocally drives HECTD1 transcription, establishing a positive regulatory loop that sustains glycolysis and Th17 differentiation. This circuit promotes intestinal inflammation and colitis‐associated cancer, highlighting HECTD1 as a potential therapeutic target for IBD and CAC.

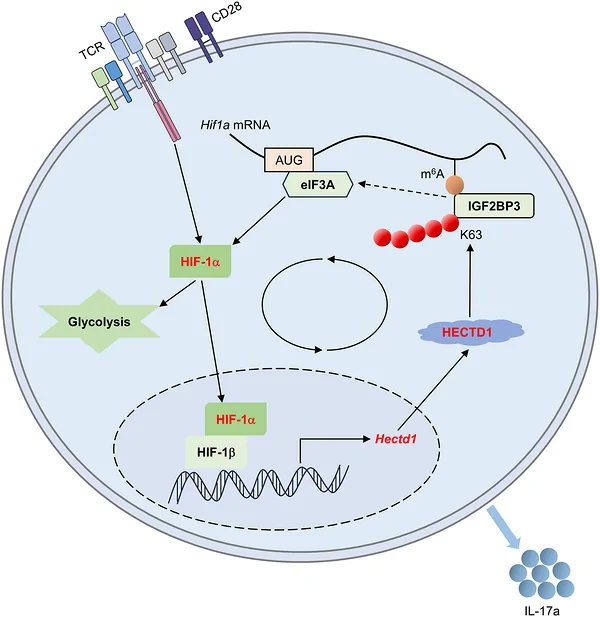

## 1 Introduction

Inflammatory bowel disease (IBD) encompasses chronic relapsing disorders of the gastrointestinal tract, which are characterized by noninfectious intestinal inflammation and can lead to severe complications, including intestinal haemorrhage, stenosis, perforation, fistulas, and toxic megacolon [1, 2, 3]. Moreover, patients with IBD are at increased risk of colorectal cancer, as chronic intestinal inflammation induces DNA damage and tumorigenesis, which results in colitis‐associated cancer (CAC) via the inflammation‐dysplasia‐carcinoma sequence [4, 5, 6, 7]. At present, therapies for IBD and CAC are relatively limited, and the precise aetiologies of these diseases are still unclear [8, 9, 10].

IL‐17‐producing T‐helper (Th17) cells, which are recognized as an important T‐cell subset, are originally differentiated from naïve CD4^+^ T cells. Th17 cells are indispensable for intestinal immune homeostasis and may contribute critically to the development and progression of IBD and CAC [11, 12, 13, 14, 15]. The differentiation and function of Th17 cells are tightly dependent on cellular glucose metabolism, especially aerobic glycolysis [16, 17, 18]. A greater glycolytic capacity and activity are observed in Th17 cells than in other subsets of CD4^+^ T cells [19]. The blockade of glycolysis inhibits Th17 cell generation and ameliorates autoimmune‐mediated inflammation [20, 21, 22]. Consequently, targeting the glucose metabolism in Th17 cells may offer therapeutic benefits for patients with IBD and CAC. Nevertheless, the molecular mechanisms orchestrating metabolic reprogramming in Th17 cells remain obscure.

Evidence implicates E3 ubiquitin ligases in modulating Th17 cell biology. HECTD3 promotes the pathogenic Th17 lineage by controlling ubiquitination of STAT3 and MALT1 [23]. TRIM21 attenuates Th17 cell differentiation by negatively modulating IRF3 expression [24]. The E3 ubiquitin ligase von Hippel–Lindau (VHL), which targets HIF‐1α for proteasomal degradation, has also been shown to promote Th17 cell differentiation and experimental autoimmune encephalomyelitis [25]. Moreover, E3 ligases have been shown to be involved in IBD and CAC progression. MARCH2 regulates TNF‐α‐mediated colitis by autoubiquitination [26]. FBXW7 targets the EZH2‐Ccl2/Ccl7 axis in macrophages to exacerbate intestinal inflammation [27]. RNF183 contributes to the development of IBD by ubiquitinating IκBα and activating the NF‐κB pathway [28]. Itch mediates ROR‐γt ubiquitination and degradation, thereby inhibiting IL‐17‐mediated intestinal inflammation and tumorigenesis [29]. Peli3 enhances TLR4 signalling and aggravates CAC via the ubiquitination‐dependent degradation of IRF4 [30]. Together, these studies establish an important role for E3 ubiquitin ligases in Th17 differentiation and intestinal inflammation. However, how ubiquitin‐dependent regulation is linked to the metabolic programming of Th17 cells remains incompletely understood.

Although HIF‐1α‐driven glycolysis is a well‐established metabolic program supporting Th17 cell differentiation, the mechanisms controlling HIF‐1α translation during this process remain incompletely defined. In particular, whether E3 ubiquitin ligases regulate HIF‐1α translation by modulating RNA‐binding proteins in Th17 cells remains unclear. Here, we investigated the role of HECTD1 in Th17 cell differentiation and metabolism and its contribution to intestinal inflammation and CAC. Using T‐cell‐specific *Hectd1* knockout mice, murine models of colitis and CAC, and in vitro Th17 differentiation, together with transcriptomic, metabolomic, and molecular analyses, we examined how HECTD1 regulates Th17 cell metabolism and HIF‐1α expression.

## 2 Results

### 2.1 HECTD1 Expression in CD4^+^ T cells Is Positively Correlated with IBD and CAC Progression

To identify the critical E3 ligases that contribute to colitis‐associated tumorigenesis, we examined the differentially expressed genes (DEGs) in CAC tissue vs. normal tissue by using the Gene Expression Omnibus (GEO) datasets (GSE124029, GSE44904, and GSE249124) for mouse CAC and the murine E3 ubiquitin ligase‐encoding genome. A Venn diagram analysis revealed 21 overlapping genes, including *Hectd1* (Figure 1A). On the basis of another GEO dataset (GSE37283) for human ulcerative colitis (UC) and CAC, we found that *HECTD1* was the most significantly upregulated gene among 21 overlapping genes in human UC and CAC tissues compared with that in normal tissue (Figure 1B). Uniform manifold approximation and projection (UMAP) analysis of an open‐access single‐cell transcriptional dataset for human colonic immune cells [31] revealed synchronous distributions between *HECTD1* transcriptional activity and CD4^+^ T‐cell subsets, especially the CD4^+^ Th17 cell subset (Figure 1C), indicating that increased expression of *HECTD1* was associated with CD4^+^ T‐cell subsets of human colonic immune cells. We also determined the mRNA and protein levels of HECTD1 in different CD4^+^ T cell subsets. Consistently, qRT‐PCR analysis and Western blotting showed that HECTD1 exhibited the highest expression level in differentiated non‐pathogenic Th17 (npTh17) and pathogenic Th17 (pTh17) cells (Figure 1D,E and Figure S1A). To investigate the function of HECTD1 in CD4^+^ T cells, we crossed *Hectd1* ^fl/fl^ mice with *Cd4*‐Cre mice to obtain *Hectd1* T‐cell‐conditional knockout (*Hectd1* ^fl/fl^ *Cd4*‐Cre) mice (Figure S1B,C). To elucidate the role of HECTD1‐deficient CD4^+^ T cells in the pathogenesis of IBD and CAC, we employed two complementary mouse models. The dextran sulphate sodium (DSS)‐induced acute colitis model was used to assess acute intestinal inflammation, whereas the DSS/azoxymethane (AOM)‐induced CAC model combines AOM‐initiated mutagenesis with DSS‐driven intestinal inflammation to model inflammation‐associated colorectal tumorigenesis. Indeed, compared with *Hectd1* ^+/+^ *Cd4*‐Cre mice, *Hectd1* ^fl/fl^ *Cd4*‐Cre acute colitis model mice exhibited significantly decreased body weight loss (Figure 1F), improved disease scores (Figure S1D), and reduced colon length shortening (Figure 1G). A mini‐endoscopic examination revealed mitigated bleeding in *Hectd1* ^fl/fl^ *Cd4*‐Cre mice compared with wild‐type (WT) *Hectd1* ^+/+^ *Cd4*‐Cre mice (Figure S1E). Hematoxylin and eosin (HE) staining further revealed that DSS treatment damaged the colonic mucosa and increased inflammatory cell infiltration, whereas *Hectd1* ^fl/fl^ *Cd4*‐Cre mice showed reduced colonic inflammatory cell infiltration compared with wild‐type (WT) *Hectd1* ^+/+^ *Cd4*‐Cre mice (Figure S1F). Consistently, in the CAC model, fewer and smaller colorectal tumours were observed in *Hectd1* ^fl/fl^ *Cd4*‐Cre mice compared with *Hectd1* ^+/+^ *Cd4*‐Cre mice (Figure 1H and Figure S1G,H). Additionally, histologic analysis showed a significant reduction in the development of hyperplasia and adenoma in the colon of *Hectd1* ^fl/fl^ *Cd4*‐Cre mice (Figure S1I). Collectively, these results suggest that HECTD1 expression in CD4^+^ T cells is positively correlated with IBD and CAC progression.

**FIGURE 1:**
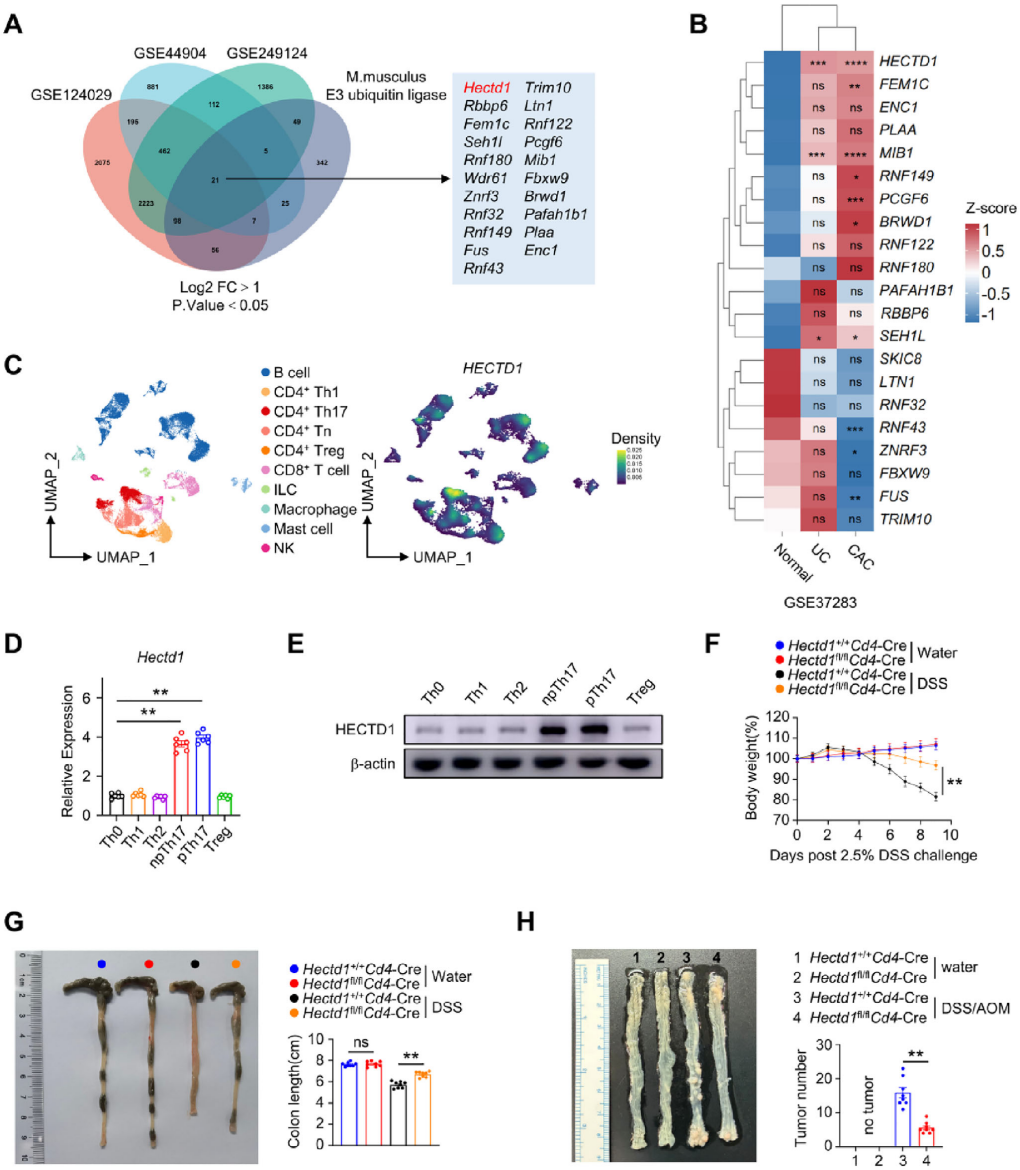
HECTD1 expression in CD4^+^ T cells is positively correlated with IBD and CAC progression. (A) Venn diagram analysis of overlapping genes between colitis‐associated colorectal cancer (CAC) vs. normal tissue differentially expressed genes (DEGs) (integrated from three GEO datasets: GSE124029, GSE44904, and GSE249124) and the murine E3 ubiquitin ligase‐encoding genome. (B) Heatmap of overlapping gene expression in human normal tissue, ulcerative colitis (UC) tissue, and CAC tissue from a GEO dataset (GSE37283). (C) Uniform manifold approximation and projection (UMAP) analysis of indicated cell subsets from human colonic immune cells (left). UMAP analysis of single‐cell transcriptional levels of HECTD1 in human colonic immune cells (right). Data source: https://www.gutcellatlas.org/tcr‐bcr. (D) Reverse‐transcriptase quantitative PCR (qRT‐PCR) analysis of *Hectd1* mRNA expression levels in CD4^+^ T cell subsets that were differentiated in vitro (n = 6). (E) Immunoblot (IB) analysis of HECTD1 in CD4^+^ T cell subsets that were differentiated in vitro. (F and G) 8‐week‐old *Hectd1* ^+/+^ *Cd4*‐Cre and *Hectd1* ^fl/fl^ *Cd4*‐Cre mice were provided with regular water or drinking water containing 2.5% DSS for 7 days, followed by regular water. Mice body weight loss (F; n = 8), representative photographs of colons and colon length (G; n = 8) were analyzed. (H) 8‐week‐old *Hectd1* ^+/+^ *Cd4*‐Cre and *Hectd1* ^fl/fl^ *Cd4*‐Cre mice received a single intraperitoneal (i.p.) injection of AOM followed by three cycles of 2.5% DSS drinking water exposure. Mice were sacrificed at day 80. Representative photographs of colorectal tumors and macroscopic colorectal tumor number (H; n = 8) were analyzed. Data are representative of three independent experiments (D‐H). Data are presented as mean ± SEM. ns, not statistically significant; **, P < 0.01. Two‐way ANOVA with Tukey's multiple comparisons (B), One‐way ANOVA with Tukey's multiple comparisons (D and G), Two‐way ANOVA with Geisser‐Greenhouse correction (F), or Two‐tailed Student's t test (H).

### 2.2 HECTD1 Promotes Th17 Cell Differentiation

To further explore the importance of HECTD1 in CD4^+^ T cells, we examined phenotypes of cells from *Hectd1* ^+/+^ *Cd4*‐Cre and *Hectd1* ^fl/fl^ *Cd4*‐Cre mice in in vitro culture. No significant anomalies in thymocyte or peripheral T‐cell development and homeostasis and in CD4^+^ T‐cell cytokine secretion, activation, proliferation, or apoptosis were detected in six‐week‐old *Hectd1* ^fl/fl^ *Cd4*‐Cre mice compared with WT mice (Figure S2A‐F). We then performed RNA sequencing (RNA‐seq) profiling of peripheral CD4^+^ T cells from *Hectd1* ^+/+^ *Cd4*‐Cre and *Hectd1* ^fl/fl^ *Cd4*‐Cre mice, which were stimulated with anti‐CD3 and anti‐CD28 antibodies for 24 h. Kyoto Encyclopedia of Genes and Genomes (KEGG) pathway enrichment analysis revealed significant differences in several important functional pathways, including gene expression related to Th17 cell differentiation, IBD, and glycolysis/gluconeogenesis, in HECTD1‐sufficient vs. ‐deficient CD4^+^ T cells (Figure 2A). In parallel, we found via a GEO dataset for UC (GSE177044) that HECTD1 expression was significantly related to Th17 cell differentiation‐associated pathways and Th17 cell pathogenic signatures (Figure 2B). Similarly, Spearman's correlation analysis of the data from healthy individuals from the Genotype‐Tissue Expression (GTEx) database [32] revealed a positive correlation between *HECTD1* expression and Th17 cell signatures, including those of pathogenic and nonpathogenic Th17 cells (Figure S2H). Importantly, we also found a significant positive correlation between *HECTD1* expression and *IL17A* expression in human IBD and CAC samples (Figure 2C). Consistent with the results of the transcriptomic analysis, expression of RORγt, a key transcription factor for Th17 cells, was decreased and Th17 cell differentiation was substantially inhibited in *Hectd1* ^fl/fl^ *Cd4*‐Cre mice (Figure 2D,E), whereas Th1 or regulatory T (Treg) cell differentiation was not altered (Figure S2G). The relative expression levels of Th17 cell signature genes were also decreased in *Hectd1* ^fl/fl^ *Cd4*‐Cre mice after Th17‐polarized differentiation in vitro (Figure S2I). A considerable reduction in the Th17 cell proportion was observed in *Hectd1* ^fl/fl^ *Cd4*‐Cre mice from both the DSS‐induced acute colitis model and the DSS/AOM‐induced CAC model (Figure 2F‐I and Figure S3A), whereas Th1, Treg, and Macrophage cell infiltration was similar to that in WT mice (Figure S2J,K,M, and N). We also distinguished GM‐CSF^+^IL‐17A^+^ and IFN‐γ^+^IL‐17A^+^ cells as pathogenic Th17‐like populations, and IL‐10^+^IL‐17A^+^ cells as a non‐pathogenic counterpart. Pathogenic Th17 cell proportion was reduced in *Hectd1* ^fl/fl^ *Cd4*‐Cre mice in both models. While non‐pathogenic Th17 cell proportions did not differ between *Hectd1* ^fl/fl^ *Cd4*‐Cre and WT mice in the DSS‐induced acute colitis model, a relative increase was observed in *Hectd1* ^fl/fl^ *Cd4*‐Cre mice in the DSS/AOM‐induced CAC model (Figure S2L,O). Consistently, these phenotypes were reproduced when *Hectd1* ^fl/fl^ mice were used as controls, with T‐cell‐specific *Hectd1* deletion attenuating acute colitis, suppressing colitis‐associated tumorigenesis, and reducing Th17 responses (Figure S3B‐H). Concomitantly, treatment with an anti‐IL17A neutralizing antibody eliminated the alleviating effect of HECTD1 deficiency on T cells from the DSS/AOM‐induced CAC model of *Hectd1* ^fl/fl^ *Cd4*‐Cre mice (Figure 2J). These data indicate that HECTD1 promotes Th17 cell differentiation, thereby driving the progression of IBD and CAC.

**FIGURE 2:**
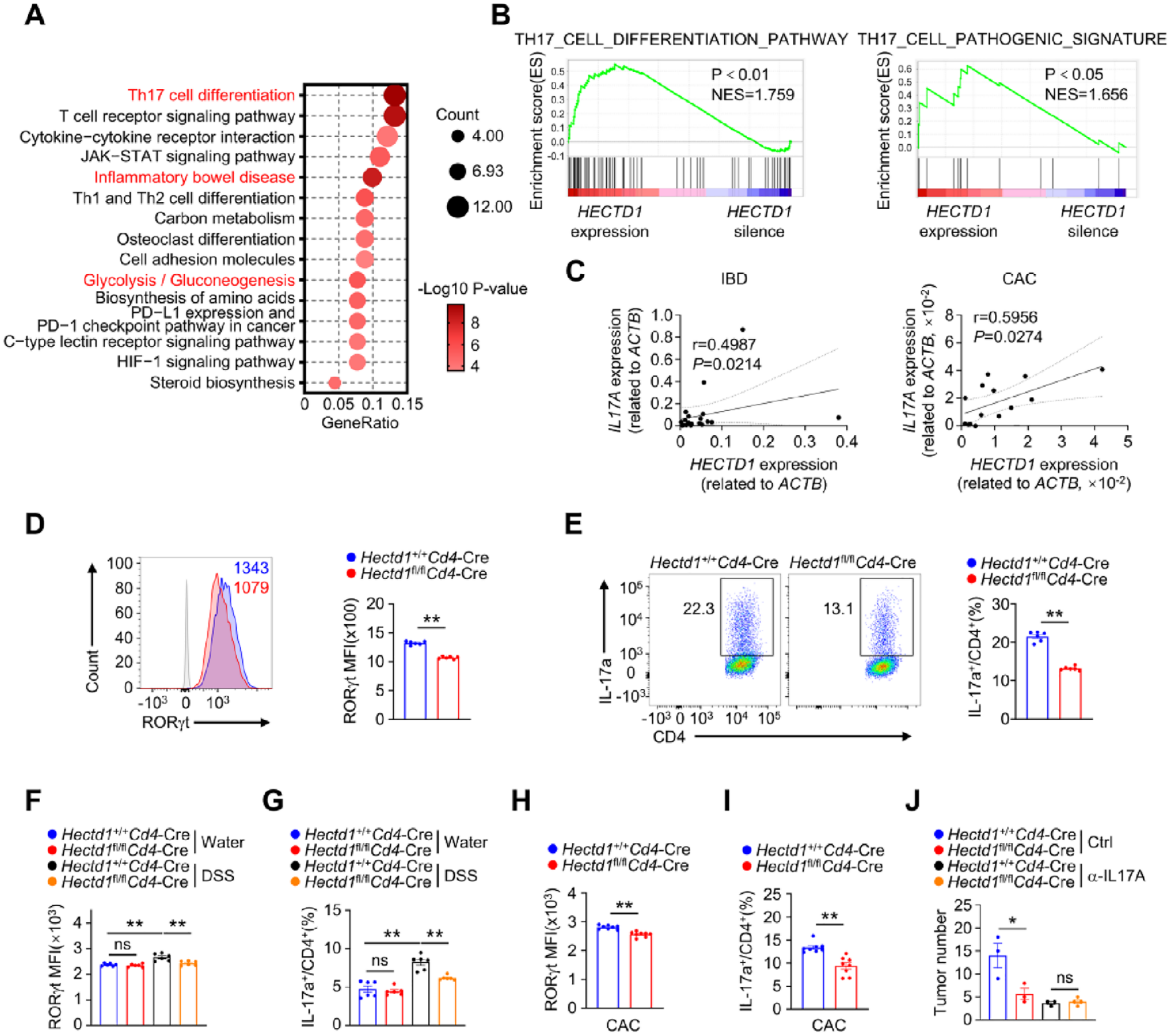
HECTD1 promotes Th17 cell differentiation. (A) Functional enrichment analysis of Kyoto Encyclopedia of Genes and Genomes (KEGG) pathways regulated in CD4^+^ T cells from *Hectd1* ^+/+^ *Cd4*‐Cre mice compared with those from *Hectd1* ^fl/fl^ *Cd4*‐Cre mice. (B) Gene set enrichment analysis (GSEA) of Th17 cell differentiation pathway‐associated genes (left) and Th17 cell pathogenic signature genes (right) in HECTD1‐expressing and HECTD1‐silenced cells from a GEO dataset for UC (GSE177044). NES, normalization enrichment score. (C) Spearman's correlation of mRNA expression of *HECTD1* and *IL17A* in tissues of IBD patients (left; n = 21) and CAC patients (right; n = 14). (D) Representative data (left) and summary graph (right) of flow cytometric analysis of the expression levels of RORγt in CD4^+^ T cells from 6‐week‐old *Hectd1* ^+/+^ *Cd4*‐Cre and *Hectd1* ^fl/fl^ *Cd4*‐Cre mice after Th17‐polarized differentiation in vitro (n = 6). (E) Representative data (left) and summary graph (right) of flow cytometric analysis of the percentages of IL‐17a‐producing CD4^+^ T cells from 6‐week‐old *Hectd1* ^+/+^ *Cd4*‐Cre and *Hectd1* ^fl/fl^ *Cd4*‐Cre mice after Th17‐polarized differentiation in vitro (n = 6). (F and G) Summary graph of flow cytometric analysis of the expression levels of RORγt in CD4^+^ T cells (F) and the percentages of IL‐17a‐producing CD4^+^ T cells (G) in the lamina propria lymphocytes (LPLs) from *Hectd1* ^+/+^ *Cd4*‐Cre and *Hectd1* ^fl/fl^ *Cd4*‐Cre mice in the DSS‐induced colitis model (n = 6). (H and I) Summary graph of flow cytometric analysis of the expression levels of RORγt in CD4^+^ T cells (H) and the percentages of IL‐17a‐producing CD4^+^ T cells (I) in the tumor‐infiltrating lymphocytes (TILs) from *Hectd1* ^+/+^ *Cd4*‐Cre and *Hectd1* ^fl/fl^ *Cd4*‐Cre mice in DSS/AOM‐induced CAC model (n = 8). (J) 8‐week‐old *Hectd1* ^+/+^ *Cd4*‐Cre and *Hectd1* ^fl/fl^ *Cd4*‐Cre mice received the DSS/AOM treatment, followed by intraperitoneal injection with immunoglobulin G control (Ctrl) or anti‐IL17A antibody (α‐IL17A). Mice were sacrificed at day 80. Macroscopic colorectal tumor numbers (n = 3 or 4) were analyzed. Data are representative of three (D‐I) independent experiments and are presented as mean ± SEM. ns, not statistically significant; *, P < 0.05; **, P < 0.01. Two‐tailed Student's *t* test (D, E, H, and I) or One‐way ANOVA with Tukey's multiple comparisons (F, G, and J).

### 2.3 HECTD1 Deletion in CD4^+^ T Cells Disrupts Glycolytic Metabolism

Since our analysis of transcriptional programs revealed differential pathways related to glycolysis (Figure 2A), we performed in‐depth mining of transcriptomic data and identified a number of glycolysis‐associated genes, including *Tpi1*, *Pgk1*, *Eno1*, *Pkm*, *Gapdh*, *Gpi1*, and *Pfkl*, which were downregulated in HECTD1‐deficient CD4^+^ T cells (Figure 3A). qRT‐PCR analysis confirmed the decreased expression of these genes in *Hectd1* ^fl/fl^ *Cd4*‐Cre Th17 cells (Figure 3B and Figure S3I). We next performed metabolomic profiling to determine whether these transcriptional changes were accompanied by alterations in cellular metabolism. HECTD1 deficiency resulted in broadly decreased levels of glycolytic metabolites, including glucose‐6‐phosphate (G6P), fructose‐6‐phosphate (F6P), fructose‐1,6‐bisphosphate (F1,6BP), dihydroxyacetone phosphate (DHAP), glyceraldehyde‐3‐phosphate (G3P), phosphoenolpyruvate (PEP), and lactate, further supporting impaired glycolytic metabolism (Figure 3C). Consistent with these findings, HECTD1‐deficient CD4^+^ T cells presented lower glucose uptake (Figure 3D) and lower glycolytic rates (Figure 3E,F). To confirm the restraining effect of glycolysis suppression on Th17 cell differentiation, Th17 cell differentiation was studied in vitro in the presence of 2‐deoxy‐D‐glucose (2‐DG), a glycolysis inhibitor. The results showed that 2‐DG treatment limited Th17 polarization and eliminated the difference between HECTD1‐deficient and WT CD4^+^ T cells (Figure 3G). Therefore, HECTD1 deletion in CD4^+^ T cells disrupts glycolytic metabolism and restricts Th17 cell differentiation.

**FIGURE 3:**
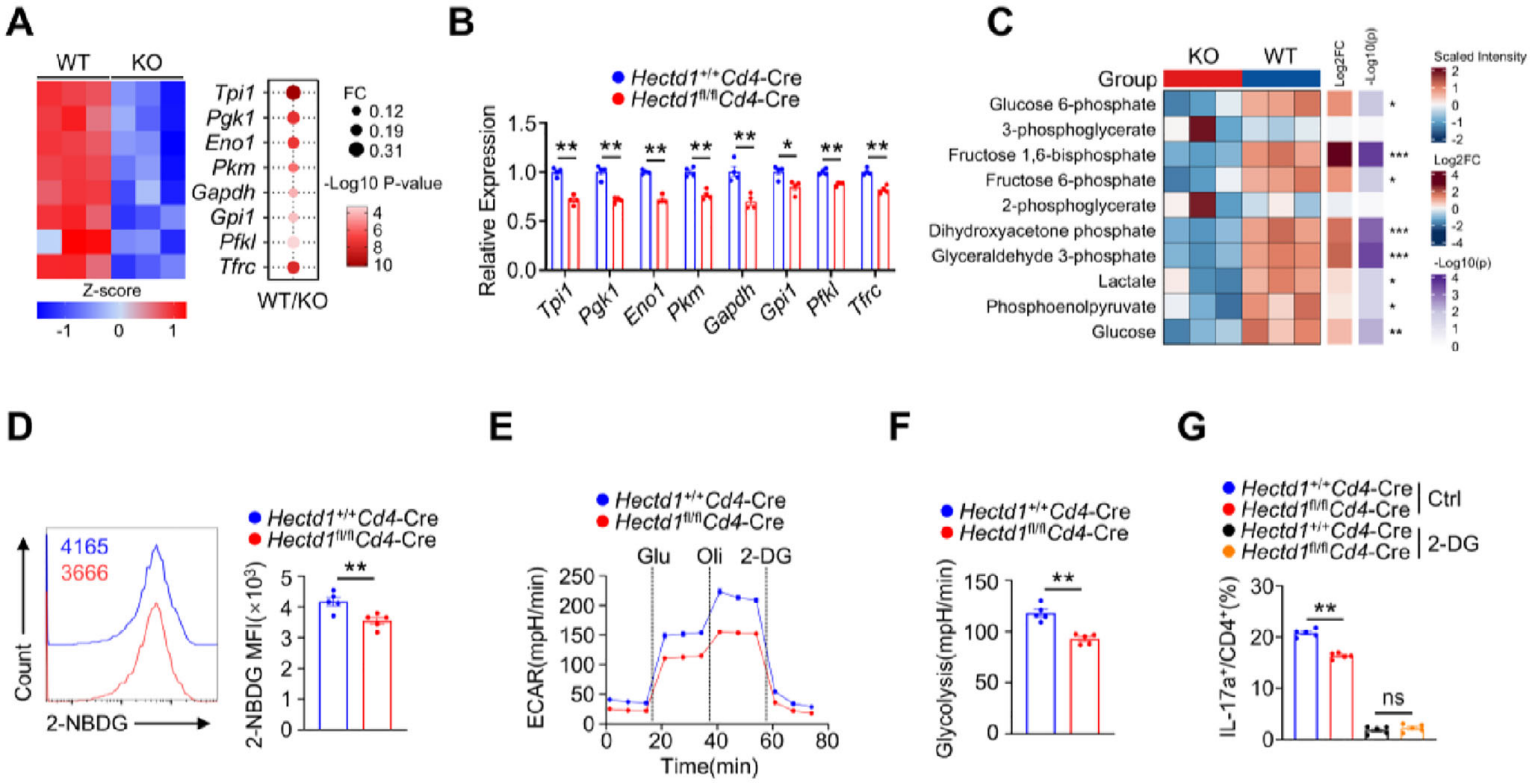
HECTD1 deletion in CD4^+^ T cells disrupts glycolytic metabolism. (A) Heatmap of upregulated genes associated with glycolysis or HIF‐1α regulation in *Hectd1* ^+/+^ *Cd4*‐Cre (WT) CD4^+^ T cells compared with those in *Hectd1* ^fl/fl^ *Cd4*‐Cre (KO) CD4^+^ T cells stimulated with α‐CD3/α‐CD28 for 24h. (B) qRT‐PCR analysis of glycolysis‐related or HIF‐1α‐regulated gene mRNA expression levels in *Hectd1* ^+/+^ *Cd4*‐Cre and *Hectd1* ^fl/fl^ *Cd4*‐Cre Th17 cells (n = 4). (C) Targeted metabolomic analysis of α‐CD3/CD28‐activated CD4^+^ T cells from *Hectd1* ^+/+^ *Cd4*‐Cre (WT) and *Hectd1* ^fl/fl^ *Cd4*‐Cre (KO) mice. The heatmap shows the scaled intensity of the indicated metabolites across three independent biological replicates per group (n = 3). The columns on the right indicate the log2 fold change (log2FC) and −log10(P) for WT vs. KO cells. (D) Representative data (left) and summary graph (right) of flow cytometric detection of 2‐NBDG uptake (30 min) in *Hectd1* ^+/+^ *Cd4*‐Cre and *Hectd1* ^fl/fl^ *Cd4*‐Cre CD4^+^ T cells stimulated with α‐CD3/α‐CD28 for 72h (n = 5). (E) Extracellular acidification rate (ECAR) of differentiated Th17 cells in vitro from *Hectd1* ^+/+^ *Cd4*‐Cre and *Hectd1* ^fl/fl^ *Cd4*‐Cre mice (n = 5). (F) Summary graph of glycolysis levels of differentiated Th17 cells as in (E) (n = 5). (G) Summary graph of flow cytometric analysis of the percentages of IL‐17a‐producing CD4^+^ T cells from 6‐week‐old *Hectd1* ^+/+^ *Cd4*‐Cre and *Hectd1* ^fl/fl^ *Cd4*‐Cre mice after Th17‐polarized differentiation in vitro in the presence of 2 mM 2‐DG (n = 5). Data are representative of three independent experiments (B, D‐G) and are presented as mean ± SEM. ns, not statistically significant; *, P < 0.05; **, P < 0.01; ***, P < 0.001. Two‐tailed Student's *t* test (B‐D and F) or one‐way ANOVA with Tukey's multiple comparisons (G).

### 2.4 HECTD1 Depends on HIF‐1α to Promote Th17 Cell Differentiation, Glycolytic Metabolism, and IBD Progression

Hypoxia‐inducible factor‐1α (HIF‐1α) is known to mediate glucose metabolism and differentiation of Th17 cells [33]. Notably, the mRNA levels of genes that are transcriptionally regulated by HIF‐1α were significantly lower in *Hectd1* ^fl/fl^ *Cd4*‐Cre Th17 cells than in *Hectd1* ^+/+^ *Cd4*‐Cre Th17 cells (Figure 3B and Figure S3I). Consistently, the protein level of HIF‐1α was also significantly lower in activated HECTD1‐deficient CD4^+^ T cells than in activated WT CD4^+^ T cells (Figure 4A and Figure S4A). To define the pivotal role of HIF‐1α in HECTD1‐mediated Th17 cell differentiation, glycolytic metabolism, and IBD progression, we performed both in vitro and in vivo experiments in which we either increased HIF‐1α expression (via overexpression or the agonist ML228) or suppressed it using the inhibitor PX‐478. Both HIF‐1α overexpression and pharmacological activation promoted glycolytic rates (Figure 4B‐E) and Th17 polarization efficiency (Figure 4H,I), and abolished the differences between HECTD1‐deficient and WT CD4^+^ T cells. Consistently, pharmacological inhibition of HIF‐1α suppressed glycolytic rates (Figure 4F,G) and Th17 polarization efficiency (Figure 4J) and also eliminated the differences between HECTD1‐deficient and WT CD4^+^ T cells. Furthermore, administration of ML228 exacerbated the severity of colitis in the *Hectd1* ^fl/fl^ *Cd4*‐Cre mice and eliminated the differences between *Hectd1* ^+/+^ *Cd4*‐Cre and *Hectd1* ^fl/fl^ *Cd4*‐Cre mice (Figure 4K‐M and Figure S4B‐D), with no statistically significant differences in colon length (Figure 4L), colonic inflammatory cell infiltration (Figure S4B), or the infiltration of total, pathogenic, and non‐pathogenic Th17 cells within lamina propria lymphocytes (LPLs) (Figure 4M and Figure S4C,D). In addition, in the presence of PX‐478, the relative alleviation of colitis that was caused by HECTD1 deficiency in CD4^+^ T cells was eliminated (Figure S4F‐H), as both *Hectd1* ^+/+^ *Cd4*‐Cre and *Hectd1* ^fl/fl^ *Cd4*‐Cre mice presented longer colon lengths (Figure S4G), less Th17 cell infiltration in the LPLs (Figure S4H), and abated bleeding of the colon (Figure S4F). No differences in the proportions of Th1 or Treg cells in the DSS‐induced acute colitis model (Figure S4E,H). These results suggest that HECTD1 depends on HIF‐1α to promote Th17 cell differentiation, glycolytic metabolism, and IBD progression.

**FIGURE 4:**
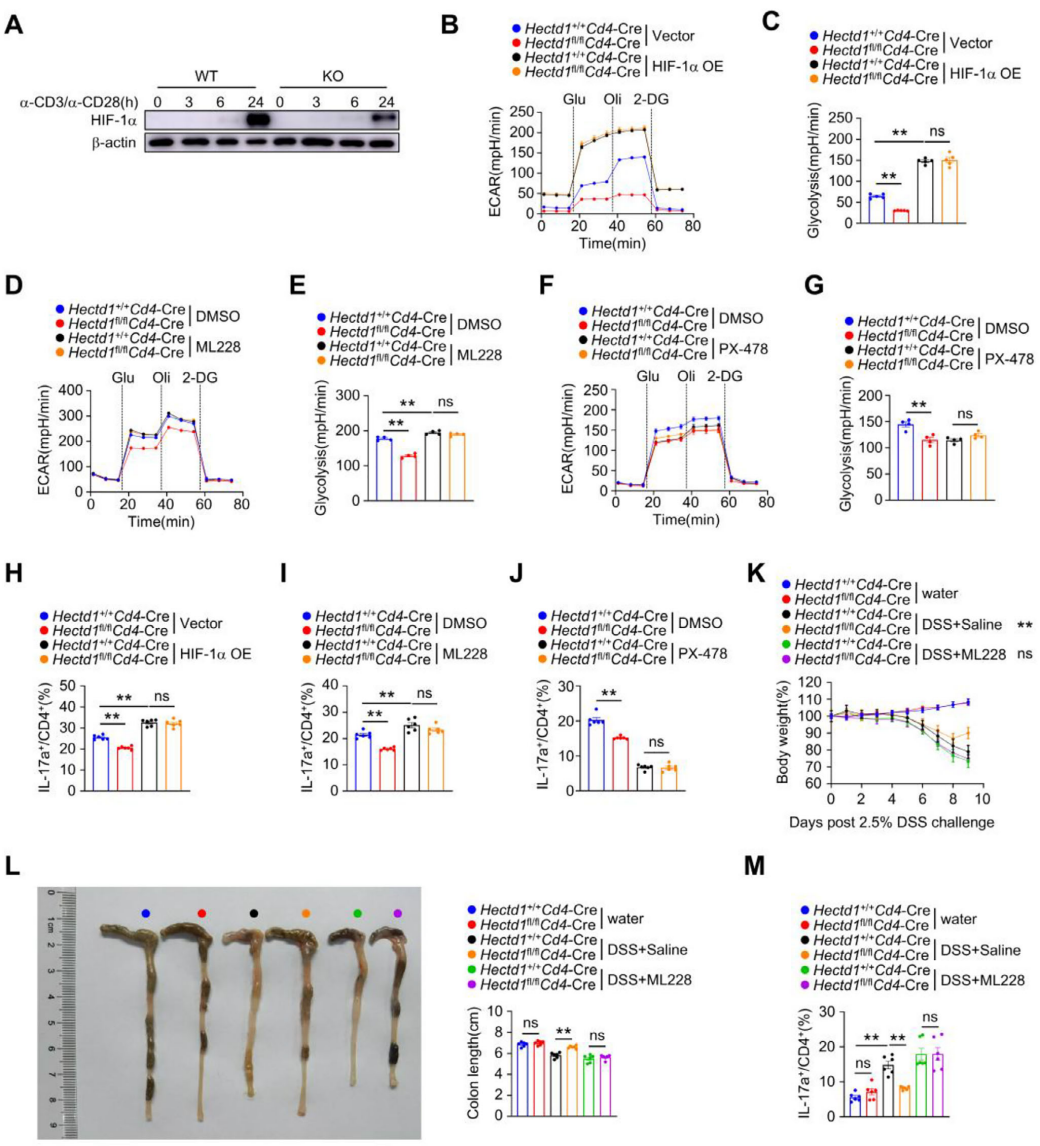
HECTD1 depends on HIF‐1α to promote Th17 cell differentiation, glycolytic metabolism, and IBD progression. (A) IB analysis of HIF‐1α in *Hectd1* ^+/+^ *Cd4*‐Cre (WT) and *Hectd1* ^fl/fl^ *Cd4*‐Cre (KO) CD4^+^ T cells stimulated with α‐CD3/α‐CD28 for 0, 3, 6, and 24h. (B) ECAR of *Hectd1* ^+/+^ *Cd4*‐Cre and *Hectd1* ^fl/fl^ *Cd4*‐Cre CD4^+^ T cells stimulated with α‐CD3/α‐CD28 for 24h and transduced with HIF‐1α or empty vector (n = 5). (C) Summary graph of glycolysis levels of CD4^+^ T cells as in (B) (n = 5). (D) ECAR of *Hectd1* ^+/+^ *Cd4*‐Cre and *Hectd1* ^fl/fl^ *Cd4*‐Cre CD4^+^ T cells stimulated with α‐CD3/α‐CD28 for 48h in the presence of 2 µM HIF‐1α agonist ML228 or DMSO as control (n = 4). (E) Summary graph of glycolysis levels of CD4^+^ T cells as in (D) (n = 4). (F) ECAR of *Hectd1* ^+/+^ *Cd4*‐Cre and *Hectd1* ^fl/fl^ *Cd4*‐Cre CD4^+^ T cells stimulated with α‐CD3/α‐CD28 for 48h in the presence of 20 µM HIF‐1α inhibitor PX‐478 or DMSO as control (n = 4). (G) Summary graph of glycolysis levels of CD4^+^ T cells as in (F) (n = 4). (H) Summary graph of flow cytometric analysis of the percentages of IL‐17a‐producing CD4^+^ T cells from 6‐week‐old *Hectd1* ^+/+^ *Cd4*‐Cre and *Hectd1* ^fl/fl^ *Cd4*‐Cre mice after transduction with HIF‐1α or empty vector and Th17‐polarized differentiation in vitro (n = 6). (I) Summary graph of flow cytometric analysis of the percentages of IL‐17a‐producing CD4^+^ T cells from 6‐week‐old *Hectd1* ^+/+^ *Cd4*‐Cre and *Hectd1* ^fl/fl^ *Cd4*‐Cre mice after Th17‐polarized differentiation in vitro in the presence of 2 µM ML228 or DMSO as control (n = 6). (J) Summary graph of flow cytometric analysis of the percentages of IL‐17a‐producing CD4^+^ T cells from 6‐week‐old *Hectd1* ^+/+^ *Cd4*‐Cre and *Hectd1* ^fl/fl^ *Cd4*‐Cre mice after Th17‐polarized differentiation in vitro in the presence of 20 µM PX‐478 or DMSO as control (n = 6). (K and L) 8‐week‐old *Hectd1* ^+/+^ *Cd4*‐Cre and *Hectd1* ^fl/fl^ *Cd4*‐Cre mice received the DSS treatment, followed by intraperitoneal (i.p.) injection with saline or 0.25 mg/kg ML228. Mice were sacrificed at day 10. Body weight loss (K) and colon length (L) were analyzed (n = 6). (M) Summary graph of flow cytometric analysis of the percentages of IL‐17a‐producing CD4^+^ T cells in the LPLs of mice as in (K) (n = 6). Data are representative of three (A‐J) or two (K‐M) independent experiments and are presented as mean ± SEM. ns, not statistically significant; **, P < 0.01. One‐way ANOVA with Tukey's multiple comparisons (C, E, G‐J, L, and M) or Two‐way ANOVA with Geisser‐Greenhouse correction (K).

### 2.5 HECTD1 Enhances IGF2BP3‐Based HIF‐1α Translation Efficiency in CD4^+^ T Cells

To determine how HIF‐1α expression is regulated by HECTD1, we first assessed the transcriptional level of *Hif1a* in HECTD1‐deficient CD4^+^ T cells. qRT‐PCR analysis revealed that HECTD1 deficiency did not affect *Hif1a* mRNA levels (Figure 5A). After the addition of the RNA synthesis inhibitor actinomycin D (Act. D) to activated CD4^+^ T cells, HECTD1 deficiency did not affect the *Hif1a* mRNA degradation rate (Figure S5A), which suggested that HECTD1 did not affect *Hif1a* mRNA stability. Then, activated CD4^+^ T cells were exposed to the proteasome inhibitor MG132 or the protein‐synthesis inhibitor cycloheximide (CHX). As a result, blockade of protein synthesis with CHX led to a considerable decrease in the HIF‐1α protein level and eliminated the significant difference between *Hectd1* ^+/+^ *Cd4*‐Cre and *Hectd1* ^fl/fl^ *Cd4*‐Cre CD4^+^ T cells (Figure 5B); however, MG132 exposure, which could impede protein degradation, failed to eliminate this difference (Figure 5B). Moreover, there was no significant difference in the HIF‐1α protein half‐life between WT and HECTD1‐deficient CD4^+^ T cells (Figure S5B), indicating that HECTD1 is dispensable for HIF‐1α protein stability. Furthermore, we evaluated whether HECTD1 modulates HIF‐1α translation by polyribosome analysis (Figure 5C and Figure S5C). Strikingly, a marked decrease in *Hif1a* mRNA translation was detected in HECTD1‐deficient CD4^+^ T cells (Figure 5C) and differentiated Th17 cells (Figure S5C), suggesting that the HIF‐1α translation efficiency is restrained by HECTD1 deletion. m^6^A methylation represents a pivotal mechanism that regulates translational efficiency, and IGF2BP3, an m^6^A reader, has been proven to preferentially recognize m^6^A‐modified mRNAs and promote the translation of potential mRNA targets [34]. m^6^A RIP analysis revealed an increased level of m^6^A modification of *Hif1a* mRNA in CD4^+^ T cells upon T cell receptor (TCR) stimulation (Figure 5D) and differentiated Th17 cells (Figure S5D), although HECTD1 deficiency did not affect the m^6^A enrichment of *Hif1a* mRNA (Figure 5D and Figure S5D). Unbiased co‐immunoprecipitation experiments revealed that HECTD1 interacts with IGF2BP3, but not with the other m^6^A reader proteins tested under our conditions (Figure S5E). This is consistent with the identification of IGF2BP3 as a candidate in previous co‐IP/MS data, though IGF2BP1 and IGF2BP2 had also been suggested as potential interactors [35]. The RIP analysis confirmed the interaction of IGF2BP3 with *Hif1a* mRNA in activated CD4^+^ T cells (Figure 5E) and differentiated Th17 cells (Figure S5F), and HECTD1 ablation inhibited this interaction (Figure 5E and Figure S5F). Recent studies have revealed that one of the key mechanisms by which m^6^A reader proteins promote translation is through their association with translation initiation modules, such as the eIF complex [36, 37]. Indeed, the interaction of IGF2BP3 with eIF3A increased upon TCR activation and was dampened by HECTD1 deletion (Figure 5F and Figure S5G,H). Accordingly, an association between eIF3A and *Hif1a* mRNA was observed in activated CD4^+^ T cells (Figure 5G) and differentiated Th17 cells (Figure S5I), and was attenuated by HECTD1 deficiency (Figure 5G and Figure S5I). Using the SRAMP database, we predicted potential m^6^A motif sites (sites 558, 1099, 1747, 1828, and 2214) on *Hif1a* mRNA that may be recognized by IGF2BP3. IGF2BP3 overexpression plasmid and either wild‐type (WT) or mutant *Hif1a* luciferase reporter plasmids were co‐transfected into HEK293T cells, followed by dual‐luciferase assays. The results showed that IGF2BP3 bound to *Hif1a* mRNA (Figure S5J). Mutations at several predicted sites (site 558, 1099, 1747, and 2214) significantly reduced the ability of IGF2BP3 to support reporter activity, whereas a mutation at site 1828 had no effect (Figure S5J). These results collectively demonstrate that HECTD1 increases IGF2BP3‐dependent m^6^A‐based HIF‐1α translation efficiency in CD4^+^ T cells.

**FIGURE 5:**
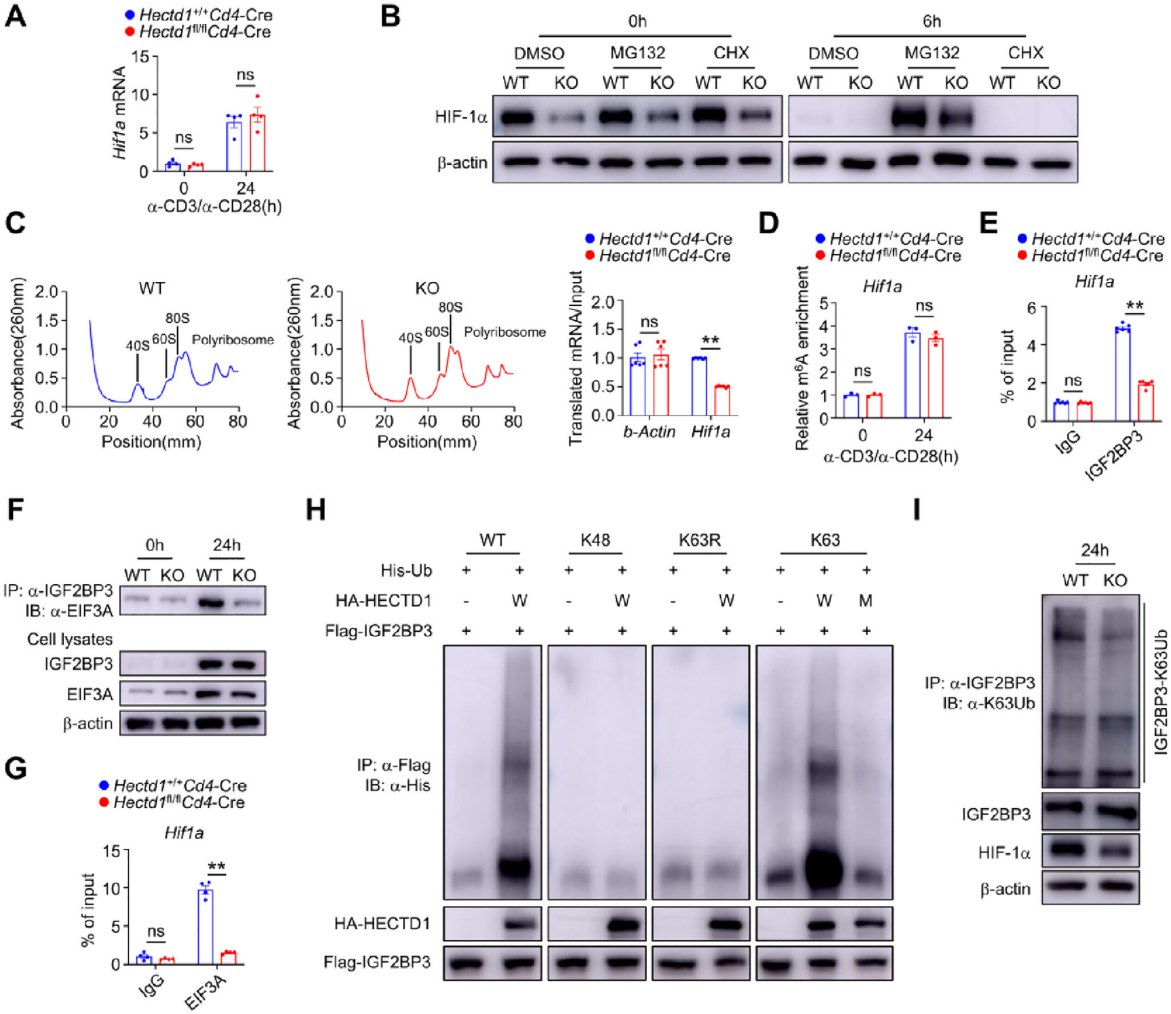
HECTD1 enhances IGF2BP3‐based HIF‐1α translation efficiency in CD4^+^ T cells. (A) qRT‐PCR analysis of *Hif1a* mRNA levels in *Hectd1* ^+/+^ *Cd4*‐Cre and *Hectd1* ^fl/fl^ *Cd4*‐Cre CD4^+^ T cells stimulated with α‐CD3/α‐CD28 for 0 or 24h (n = 4). (B) IB analysis of HIF‐1α in *Hectd1* ^+/+^ *Cd4*‐Cre (WT) and *Hectd1* ^fl/fl^ *Cd4*‐Cre (KO) CD4^+^ T cells stimulated with α‐CD3/α‐CD28 for 24h in the presence of DMSO, 20 µM MG132, and 20 µg/mL cycloheximide (CHX) for 0 or 6h. (C) Polyribosome analysis: representative traces of ribosome extract from stimulated *Hectd1* ^+/+^ *Cd4*‐Cre (WT) and *Hectd1* ^fl/fl^ *Cd4*‐Cre (KO) CD4^+^ T cells in the presence of 100 µg/mL CHX (left); qRT‐PCR analysis of relative expression ratio of polyribosome mRNAs to input mRNAs after sucrose gradient fraction of polyribosomes (right; n = 6). (D) m^6^A‐RIP‐qRT‐PCR analysis of m^6^A levels of *Hif1a* mRNA in *Hectd1* ^+/+^ *Cd4*‐Cre and *Hectd1* ^fl/fl^ *Cd4*‐Cre CD4^+^ T cells stimulated with α‐CD3/α‐CD28 for 0 or 24h (n = 3). (E) RIP analysis of the interaction of IGF2BP3 with *Hif1a* mRNA in *Hectd1* ^+/+^ *Cd4*‐Cre and *Hectd1* ^fl/fl^ *Cd4*‐Cre CD4^+^ T cells stimulated with α‐CD3/α‐CD28 for 24h (n = 6). (F) Lysates from *Hectd1* ^+/+^ *Cd4*‐Cre (WT) and *Hectd1* ^fl/fl^ *Cd4*‐Cre (KO) CD4^+^ T cells stimulated with α‐CD3/α‐CD28 for 24h were subjected to immunoprecipitation (IP) with α‐IGF2BP3 antibody. (G) RIP analysis of the interaction of eIF3A with *Hif1a* mRNA in *Hectd1* ^+/+^ *Cd4*‐Cre and *Hectd1* ^fl/fl^ *Cd4*‐Cre CD4^+^ T cells stimulated with α‐CD3/α‐CD28 for 24h (n = 4). (H) IB and IP analysis of IGF2BP3 ubiquitination in HEK293T cells co‐transfected with the indicated vectors. W, WT HECTD1; M, mutant HECTD1 C2579G lacking the E3 ligase activity. (I) IB and IP analysis of IGF2BP3 K63‐linked ubiquitination in *Hectd1* ^+/+^ *Cd4*‐Cre (WT) and *Hectd1* ^fl/fl^ *Cd4*‐Cre (KO) CD4^+^ T cells stimulated with α‐CD3/α‐CD28 for 24h. Data are representative of three independent experiments and are presented as mean ± SEM. ns, not statistically significant; **, P < 0.01. Two‐tailed Student's *t* test (A, C‐E, and G).

HECTD1 is an E3 ubiquitin ligase that directs protein ubiquitination and controls protein stability, protein–protein interactions, among other functions [38]. Different ubiquitin linkages can confer distinct functional outcomes. K48‐linked polyubiquitination is predominantly associated with proteasomal degradation, whereas K63‐linked polyubiquitination generally serves non‐proteolytic functions, including the regulation of protein interactions and signaling. Accordingly, K48‐ and K63‐only ubiquitin constructs were used to distinguish these linkage types, whereas the K63R mutant, in which Lys63 is replaced by arginine (R), prevents ubiquitin‐chain extension through K63. Immunoprecipitation (IP) and IB analysis revealed that IGF2BP3 coprecipitated with HECTD1 and with WT or K63‐ubiquitin but not with K48‐ or K63R‐ubiquitin, showing increased IGF2BP3 ubiquitination (Figure 5H), whereas the HECTD1 mutant lacking E3 ligase activity was not able to mediate IGF2BP3 ubiquitination (Figure 5H), implying that HECTD1 directs K63‐linked ubiquitination of the specific substrate IGF2BP3. Accordingly, K63‐linked ubiquitination was suppressed in activated CD4^+^ T cells from *Hectd1* ^fl/fl^ *Cd4*‐Cre mice but not in those from WT mice (Figure 5I). To assess the requirement of HECTD1 catalytic activity for K63‐linked ubiquitination of IGF2BP3, we overexpressed wild‐type HECTD1 or a HECT catalytically inactive HECTD1 mutant lacking E3 ubiquitin ligase activity in HECTD1‐deficient CD4^+^ T cells. Overexpression of wild‐type HECTD1, but not the catalytically inactive mutant, restored K63‐linked ubiquitination of IGF2BP3 (Figure S6A), promoted the interaction of IGF2BP3 with *Hif1a* mRNA and eIF3A (Figure S6C,E), and enhanced the binding between eIF3A and *Hif1a* mRNA (Figure S6G). Conversely, CRISPR‐Cas9‐mediated deletion of the HECT catalytic domain of HECTD1 in CD4^+^ T cells reduced K63‐linked ubiquitination of IGF2BP3 (Figure S6B), suppressed the binding of IGF2BP3 to *Hif1a* mRNA and eIF3A (Figure S6D,F), and diminished the association between eIF3A and *Hif1a* mRNA (Figure S6H). Furthermore, using the PhosphoSitePlus PTM Database, we predicted potential ubiquitination motifs on IGF2BP3 at residues K450, K280, K213, K440, and K465. To assess the functional relevance of these residues, we generated plasmids expressing wild‐type (WT) or mutant IGF2BP3, in which each predicted lysine (K) was individually substituted with arginine (R) to abrogate ubiquitination. Subsequent analysis revealed that HECTD1 primarily promotes K63‐linked ubiquitination of IGF2BP3 at residues K440 and K465 (Figure S6I). These results indicate that HECTD1 governs IGF2BP3 K63‐linked ubiquitination to enhance the coupling of IGF2BP3 with the eIF‐driven translation initiation module and potentiate m^6^A‐based HIF‐1α translation.

### 2.6 HECTD1 Regulates CD4^+^ T‐Cell Glycolytic Metabolism and Th17 Cell Differentiation in an IGF2BP3‐HIF‐1α Axis‐Dependent Manner

To verify the critical role of IGF2BP3 and HIF‐1α activities in HECTD1‐modulated CD4^+^ T‐cell glycolytic metabolism and Th17 cell differentiation, we performed CRISPR/Cas9‐mediated gene editing to generate *Igf2bp3*‐knockout (sg*Igf2bp3*) and *Hif1a*‐knockout (sg*Hif1a*) CD4^+^ T cells. The knockout efficiency was confirmed by IB analysis (Figure S7A,B). IGF2BP3 deletion reduced the HIF‐1α protein level (Figure 6A and Figure S7C), impeded the interaction between eIF3A and *Hif1a* mRNA (Figure 6B), and eliminated the decreases in these two parameters caused by HECTD1 deficiency in CD4^+^ T cells (Figure 6A,B and Figure S7C). The phenotypes of both IGF2BP3‐deficient and HIF‐1α‐deficient CD4^+^ T cells were similar to those of HECTD1‐deficient CD4^+^ T cells, which dampened glycolytic metabolism (Figure 6C,D), inhibited Th17 cell differentiation (Figure 6E) in vitro, and abrogated the disparities between the HECTD1‐deficient and HECTD1‐sufficient CD4^+^ T cells (Figure 6C‐E). An adoptive T‐cell transfer‐induced chronic colitis model was used to further elucidate the importance of IGF2BP3 and HIF‐1α activities in HECTD1‐mediated IBD progression in vivo. In this model, the adoptive transfer of naïve CD4^+^ T cells from *Hectd1* ^fl/fl^ *Cd4*‐Cre mice resulted in an increased body weight (Figure 6F), restricted Th17 cell infiltration in the LPLs (Figure 6G), and mitigated bleeding in the colon (Figure S7D). Both IGF2BP3 and HIF‐1α ablation eliminated the alleviating effect on colitis caused by HECTD1 deficiency in CD4^+^ T cells (Figure 6F,G and Figure S7D). No difference in the proportions of Th1 or Treg cells was detected in this adoptive T‐cell transfer‐induced chronic colitis model (Figure S7E,F). To further establish the functional relevance of IGF2BP3 and HIF‐1α in HECTD1‐mediated CAC progression in vivo, we employed a DSS/AOM‐induced CAC model combined with adoptive T‐cell transfer in *Rag1* ^−/−^ mice. Mice that received naïve CD4^+^ T cells from *Hectd1* ^+/+^ *Cd4*‐Cre mice developed the highest number of colon tumours (Figure 6H) and showed marked intratumoural Th17 cell infiltration (Figure 6I). Furthermore, deletion of either IGF2BP3 or HIF‐1α eliminated the differences in the effects caused by HECTD1 deficiency in CD4^+^ T cells in the CAC model (Figure 6H,I). These data suggest that HECTD1 regulates CD4^+^ T‐cell glycolytic metabolism and Th17 cell differentiation in an IGF2BP3‐HIF‐1α axis‐dependent manner.

**FIGURE 6:**
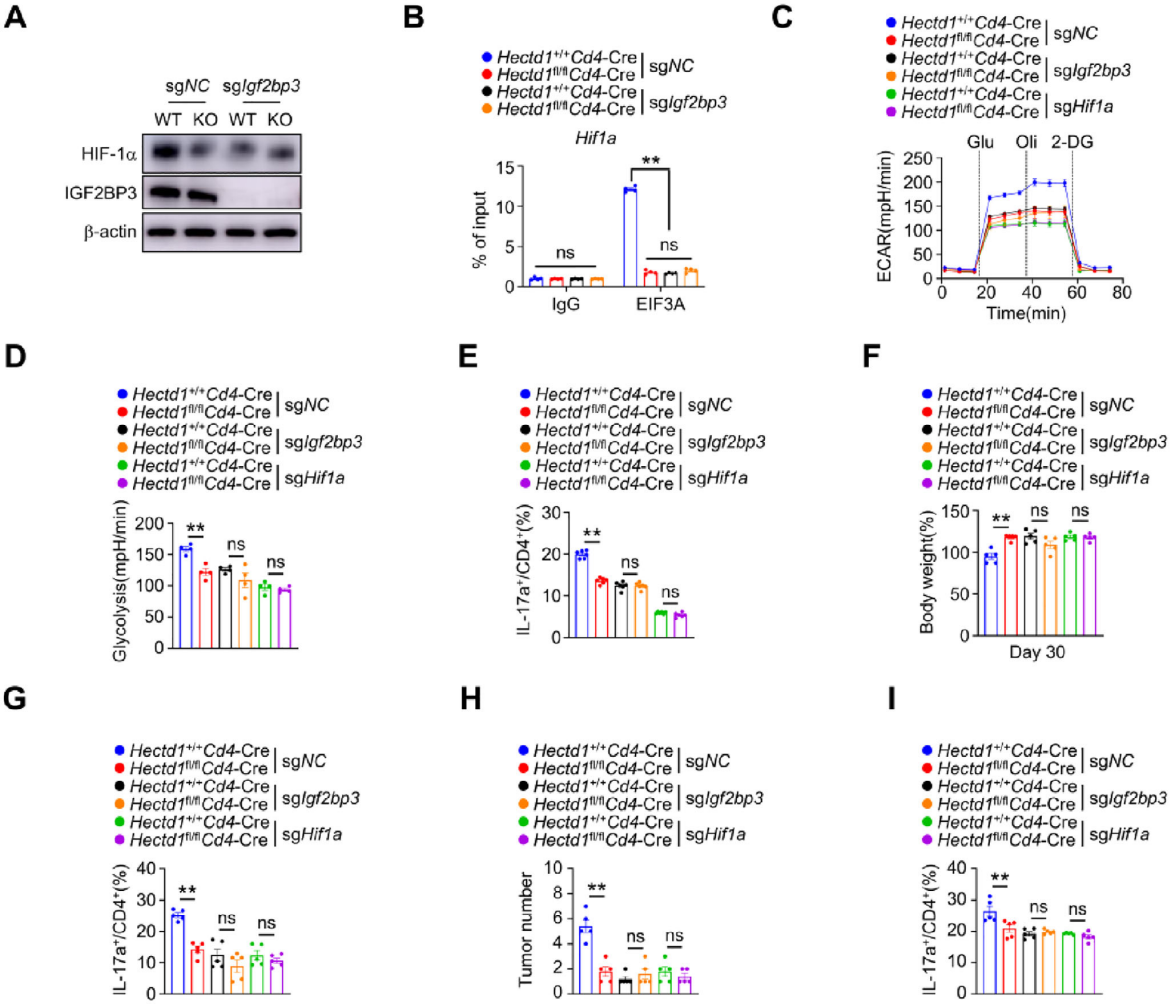
HECTD1 regulates CD4^+^ T cell glycolytic metabolism and Th17 cell differentiation in an IGF2BP3‐HIF‐1α axis‐dependent manner. (A) IB analysis of indicated proteins in *Hectd1* ^+/+^ *Cd4*‐Cre (WT) and *Hectd1* ^fl/fl^ *Cd4*‐Cre (KO) CD4^+^ T cells stimulated with α‐CD3/α‐CD28 for 24h after electro‐transfer into sg*NC* or sg*Igf2bp3*. (B) RIP analysis of the interaction of eIF3A with *Hif1a* mRNA in *Hectd1* ^+/+^ *Cd4*‐Cre and *Hectd1* ^fl/fl^ *Cd4*‐Cre CD4^+^ T cells stimulated with α‐CD3/α‐CD28 for 24h after electro‐transfer into sg*NC* or sg*Igf2bp3* (n = 4). (C) ECAR of *Hectd1* ^+/+^ *Cd4*‐Cre and *Hectd1* ^fl/fl^ *Cd4*‐Cre CD4^+^ T cells stimulated with α‐CD3/α‐CD28 for 48h after electro‐transfer into sg*NC*, sg*Igf2bp3*, or sg*Hif1a* (n = 4). (D) Summary graph of glycolysis levels of CD4^+^ T cells as in (C) (n = 4). (E) Summary graph of flow cytometric analysis of the percentages of IL‐17a‐producing CD4^+^ T cells from 6‐week‐old *Hectd1* ^+/+^ *Cd4*‐Cre and *Hectd1* ^fl/fl^ *Cd4*‐Cre mice after electro‐transfer into sg*NC*, sg*Igf2bp3*, or sg*Hif1a* and Th17‐polarized differentiation in vitro (n = 6). (F and G) Naïve CD4^+^ T cells (CD4^+^CD44^low^CD62L^high^) isolated from 6‐week‐old *Hectd1* ^+/+^ *Cd4*‐Cre and *Hectd1* ^fl/fl^ *Cd4*‐Cre mice were adoptively transferred into 8‐week‐old *Rag1* ^−/−^ mice after electro‐transfer into sg*NC*, sg*Igf2bp3*, or sg*Hif1a*. Mice were sacrificed at day 30. Body weight of *Rag1* ^−/−^ recipient mice was relative to initial weight at day 30 (F; n = 5). Summary graph of flow cytometric analysis of the percentages of IL‐17a‐producing CD4^+^ T cells in the LPLs (G; n = 5). (H and I) 8‐week‐old *Rag1* ^−/−^ mice were intraperitoneally injected with AOM one week before adoptive transfer of naïve CD4^+^ T cells (CD4^+^CD44^low^CD62L^high^) isolated from 6‐week‐old *Hectd1* ^+/+^ *Cd4*‐Cre and *Hectd1* ^fl/fl^ *Cd4*‐Cre mice following electroporation with sg*NC*, sg*Igf2bp3*, or sg*Hif1a*. Mice were then subjected to three cycles of 2% DSS administration and sacrificed on day 80. Quantification of colon tumor numbers (H; n = 5) and flow cytometric analysis of the percentages of IL‐17a‐producing CD4^+^ T cells in the TILs (I; n = 5) were analyzed. Data are representative of three independent experiments (A‐E) and are presented as mean ± SEM. ns, not statistically significant; **, P < 0.01. One‐way ANOVA with Tukey's multiple comparisons (B and D‐I).

### 2.7 HIF‐1α Transcriptionally Upregulates HECTD1 Expression

Given that HECTD1 promoted HIF‐1α expression, we next investigated the potential reciprocal regulation of HECTD1 by HIF‐1α. Notably, CRISPR–Cas9‐mediated deletion of HIF‐1α reduced HECTD1 protein expression in CD4^+^ T cells (Figure S7B), indicating that HIF‐1α may positively regulate HECTD1 expression. We therefore investigated whether this regulation occurs at the transcriptional level.

To predict the key transcription factor of HECTD1, the Database of Human Transcription Factor Targets (hTFtarget), UCSC Genome Browser (UCSC), and Gene Transcription Regulation Database (GTRD) were used. Interestingly, a Venn diagram analysis indicated that 25 overlapping transcription factors, including HIF‐1α, were predicted by the three databases (Figure 7A). Consistent with these findings, a recent HIF‐1α ChIP‐seq study also suggested HECTD1 as a potential transcriptional target of HIF‐1α  [39]. The interaction of HIF‐1α with the *Hectd1* promoter region was then confirmed in both activated CD4^+^ T cells and differentiated Th17 cells via the Cleavage Under Targets and Release Using Nuclease (CUT&RUN) assay (Figure 7B and Figure S8A) and PCR analysis (Figure 7C and Figure S8B‐D). A dual‐luciferase reporter assay revealed that overexpression of HIF‐1α led to increased transcriptional activity of the *Hectd1*‐expressing plasmid in HEK293T cells (Figure 7D). After a luciferase reporter plasmid was constructed with a *Hectd1* mutant promoter region (Figure 7E), the overexpression of HIF‐1α failed to increase the transcriptional activity of the *Hectd1*‐expressing plasmid with mutations in the promoter region (Figure 7F). Deletion of HIF‐1α in CD4^+^ T cells with an sgRNA resulted in substantially lower expression of HECTD1 at both the mRNA (Figure 7G) and protein (Figure S7B) levels. In addition, pharmacologically inhibiting the activity of HIF‐1α similarly led to reduced mRNA and protein expression of HECTD1 (Figure 7H,I). Moreover, Spearman's correlation analyses of mRNA expression in human IBD and CAC samples revealed a positive correlation between *HIF1A* and *HECTD1* expression (Figure 7J,K). Similar analyses of the data from the GTEx database and a GEO dataset for IBD (GSE193677) yielded consistent results (Figure S8E,F). These data indicate that HIF‐1α reciprocally transcriptionally upregulates HECTD1 expression.

**FIGURE 7:**
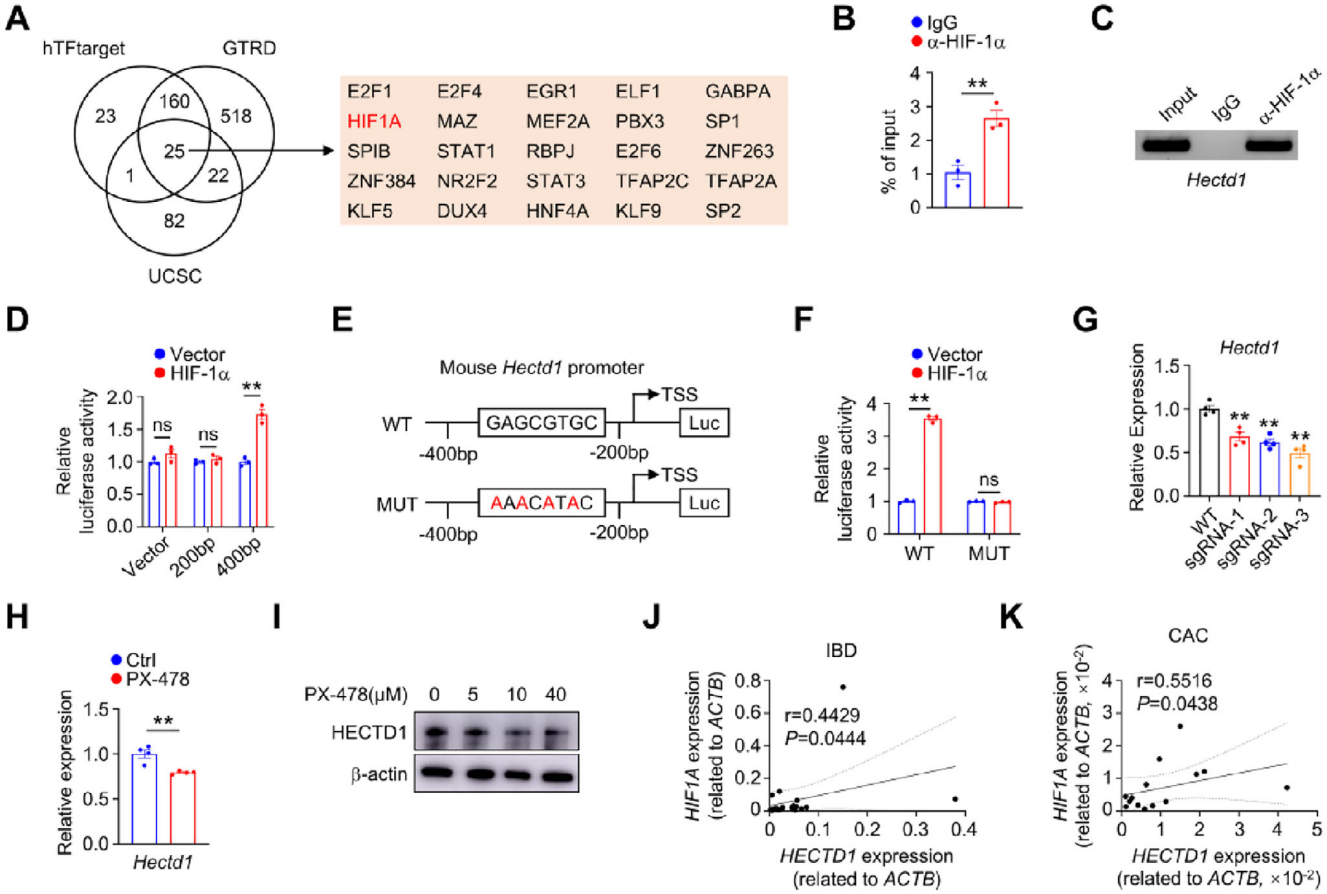
HIF‐1α transcriptionally upregulates HECTD1 expression. (A) Venn diagram showing HECTD1 transcription factors predicted by public databases hTFtarget, UCSC, and GTRD. (B and C) CUT&RUN‐qRT‐PCR (B) or PCR (C) analysis of the interaction of HIF‐1α with *Hectd1* promoter region using CD4^+^ T cells stimulated with α‐CD3/α‐CD28 for 24h (n = 3). (D) Dual luciferase reporter assay of HEK293T cells after co‐transfection with HIF‐1α overexpression plasmid and luciferase reporter plasmid containing *Hectd1* promoter region (n = 3). (E) Schematic diagram of the *Hectd1* promoter construction based on a luciferase reporter vector. WT, wildtype; MUT, mutant; Luc, luciferase. (F) Dual luciferase assay of HEK293T cells after co‐transfection with HIF‐1α overexpression plasmid and luciferase reporter plasmid containing *Hectd1* WT or MUT promoter region as in (E) (n = 3). (G) qRT‐PCR analysis of *Hectd1* mRNA levels in CD4^+^ T cells stimulated with α‐CD3/α‐CD28 for 24h after electro‐transfer into sg*NC* or sg*Hif1a* (n = 4). (H) qRT‐PCR analysis of *Hectd1* mRNA levels in CD4^+^ T cells stimulated with α‐CD3/α‐CD28 for 24h in the presence of 20 µM PX‐478 (n = 4). (I) IB analysis of HECTD1 in CD4^+^ T cells stimulated with α‐CD3/α‐CD28 for 24 h in the presence of PX‐478. (J and K) Spearman's correlation of mRNA expression of *HECTD1* and *HIF1A* in tissues of IBD patients (J; n = 21) and CAC patients (K; n = 14). Data are representative of three independent experiments (B‐I) and are presented as mean ± SEM. ns, not statistically significant; **, P < 0.01. Two‐tailed Student's *t* test (B, D, F, and H) and one‐way ANOVA with Tukey's multiple comparisons (G).

## 3 Discussion

In this study, we revealed a critical mechanism of mutual regulation between HECTD1 and HIF‐1α to orchestrate Th17 cell fate and glucose metabolism, thereby driving IBD and CAC progression. We found that HECTD1 expression was significantly correlated with IBD and CAC pathogenesis. Specific deletion of HECTD1 in T cells decreased the severity of colitis and CAC in vivo by restraining Th17 cell differentiation. RNA‐seq profiling, disease models, cellular metabolic and biochemical analyses revealed that HECTD1 deficiency decreased HIF‐1α protein expression, disrupted glycolytic metabolism, inhibited Th17 cell differentiation, and thus alleviated intestinal inflammation and tumorigenesis. Mechanistically, HECTD1 drives HIF‐1α biosynthesis via ubiquitin‐dependent IGF2BP3 modification and m^6^A‐based translational apparatus assembly. Reciprocally, HIF‐1α initiates the transcription of *Hectd1* to activate its expression, which induces a positive feedback loop, culminating in a mutual reinforcement system in CD4^+^ T cells.

HECTD1, a member of the HECT family of E3 ubiquitin ligases, which transfers ubiquitin directly to substrate proteins, has been reported to regulate embryonic development, signal transduction, ribosome assembly, and protein synthesis [35, 38, 40, 41]. Recent studies have revealed that HECTD1 is involved in the pathophysiological processes of several chronic inflammatory conditions and malignancies, such as autophagy regulation in osteoarthritis, modulation of NF‐κB pathway in colitis, angiogenesis in hepatocellular carcinoma, and ferroptosis in gastric cancer [42, 43, 44, 45]. However, the potential roles of HECTD1 in T cells and the inflammation‐to‐cancer transition are still unknown. This study supports an alternative perspective, suggesting that HECTD1 specifically regulates Th17 cell polarization via HIF‐1α‐dependent glycolytic reprogramming, which results in the development of IBD and CAC. In view of the key role of Th17 cells in autoimmune‐mediated tissue inflammation, such as rheumatoid arthritis, psoriasis, and multiple sclerosis [46], further studies are needed to determine whether HECTD1 has comparable pathogenic effects in other disease models.

As a master transcription factor of hypoxia and glycolysis, HIF‐1α modulates glucose metabolism and differentiation in Th17 cells [47, 48, 49, 50]. On the one hand, HIF‐1α governs glycolysis in Th17 cells via the transcriptional regulation of glycolytic genes and core enzymes, including glucose transporters, hexokinases, and lactate dehydrogenase [50]. On the other hand, HIF‐1α‐mediated transcriptional induction of RORγt critically orchestrates Th17 cell lineage commitment and functional activation [48]. In addition, HIF‐1α was reported to be pathologically amplified in IBD and colorectal cancer (CRC) lesion tissue, which was correlated with an advanced disease stage [51, 52]. These findings suggest that HIF‐1α serves as an inflammation‐tumorigenesis linchpin, directing malignant transition in chronically inflamed tissues. Notably, the molecular mechanisms governing mRNA modifications and the expression of HIF‐1α in Th17 cells remain elusive. The present study is the first to clarify that E3 ligase‐mediated K63‐linked ubiquitination directs the assembly of *Hif1a* mRNA with the translation initiation complex and facilitates the protein expression of HIF‐1α. Our findings clearly reveal that IGF2BP3‐dependent m^6^A modification modulates HIF‐1α activity and glycolytic metabolism in Th17 cells, thereby mediating intestinal inflammation and inflammation‐induced carcinogenesis.

Interestingly, HIF‐1α also functions as a regulator of the Treg/Th17 balance. Early studies by Dang et al. and Shi et al. demonstrated that *Hif1a* deletion suppresses Th17 differentiation while promoting Treg generation under Th17‐skewing conditions and in experimental autoimmune encephalomyelitis (EAE) models, highlighting the central role of the mTOR–HIF‐1α–glycolysis axis in Th17/Treg fate determination [20, 47]. However, subsequent studies have revealed a more nuanced role for HIF‐1α in Treg regulation. Ben‐Shoshan et al. and Clambey et al. reported that under hypoxic inflammatory conditions, HIF‐1α can promote FOXP3 expression and enhance Treg suppressive function [53, 54]. Conversely, Lee et al. showed that sustained HIF‐1α activation following Foxp3‐specific deletion of *Vhl* destabilized Treg suppressive capacity and exacerbated inflammatory pathology, whereas deletion of *Hif1a* alone in Treg cells did not significantly alter Treg proportions in the spleen, lymphoid tissues, or intestinal inflammatory sites [55]. Together, these studies underscore that the effects of HIF‐1α on Treg biology depend on the differentiation stage, inflammatory context, tissue hypoxia, and the mode of HIF‐1α perturbation. Consistent with this complexity, we found that HECTD1, acting as an activator of HIF‐1α, is predominantly expressed in Th17 cells rather than in other CD4^+^ T‐cell subsets. Consequently, *Hectd1* deficiency consistently reduced Th17 differentiation without causing a significant change in Treg frequency, both in vitro and in vivo. Notably, our in vivo models mainly involve acute colitis and CAC, where the inflammatory and hypoxic intestinal microenvironment may have complex effects on Treg biology; further investigation is needed to clarify how these microenvironmental factors influence Treg cells.

In particular, a notable phenomenon was observed in this study. Through the ubiquitination of the m^6^A reader IGF2BP3, which enhances the assembly of the translational initiation machinery, HECTD1 positively regulated HIF‐1α protein synthesis and selectively promoted Th17 cell differentiation. This process was reciprocally amplified as HIF‐1α enhanced *Hectd1* gene transcription, forming a distinct bidirectional regulatory circuit, culminating in a unique self‐reinforcing feedback mechanism in CD4^+^ T cells. Given the well‐established critical roles of HIF‐1α in diverse cellular contexts (dendritic cells, T cells, macrophages, etc.) [56], targeting HECTD1 may represent a superior therapeutic option for the treatment of IBD and CAC with substantial potential.

Several limitations of this study should be acknowledged. Although our clinical samples showed a correlation of *HECTD1* with *HIF1A* and *IL17A* in human IBD and CAC, the relatively limited cohort size restricts further stratified analyses. Larger clinical cohorts will help strengthen the clinical relevance of the HECTD1–HIF‐1α–IL‐17A axis and facilitate further evaluation of HECTD1 as a potential therapeutic target. Moreover, our findings establish the protective effects of genetic HECTD1 deficiency, whereas the therapeutic potential of pharmacologically targeting HECTD1 remains to be explored. High‐throughput screening of HECTD1‐targeting small molecules, followed by in vivo efficacy and biosafety evaluation, may facilitate the translation of this mechanism into therapeutic applications. Our mechanistic studies primarily focused on Th17 cells, in which HECTD1 functions as an amplifier of HIF‐1α‐dependent metabolic programming. Given the broad functions of HIF‐1α in the intestinal microenvironment, whether a similar HECTD1–HIF‐1α regulatory circuit operates in intestinal epithelial cells, macrophages, or other cell populations warrants further investigation using lineage‐specific models. Finally, although K440 and K465 were identified as important residues contributing to HECTD1‐mediated K63‐linked ubiquitination of IGF2BP3, additional ubiquitination sites cannot be excluded. Future Th17‐restricted IGF2BP3 K440/K465 double‐mutant models would enable more precise evaluation of the physiological contribution of these sites to the HECTD1–IGF2BP3–HIF‐1α regulatory axis in intestinal inflammation and CAC.

In conclusion, this study revealed a novel HECTD1‐HIF‐1α cross‐regulatory axis in CD4^+^ T cells. We elucidated that HECTD1 promotes m^6^A‐based HIF‐1α translation via IGF2BP3 ubiquitination and translation initiation complex assembly. Conversely, HIF‐1α functions as an essential transcription factor for *Hectd1* gene expression. This codependent interaction constitutes an autoamplification loop that increases glycolytic metabolism in CD4^+^ T cells and promotes Th17 cell polarization, thereby exacerbating intestinal inflammation and tumorigenesis. Given its role in pathogenesis, HECTD1‐targeted approaches represent promising new strategies for IBD and CAC management.

## 4 Methods

### 4.1 Human Samples

Human inflammatory bowel disease (IBD) and colitis‐associated cancer (CAC) samples from patients were collected at Ruijin Hospital, Shanghai, China. Informed consent was obtained from each patient, and the study protocol was approved by the Clinical Research Ethics Committee of Ruijin Hospital (Ethics Committee Reference Number 2022–239 and 2026–49) and complied with all relevant ethics regulations.

### 4.2 Mice

*Hectd1*‐floxed mice in C57BL/6 background were generated using a loxP targeting system. The *Hectd1*‐floxed mice were crossed with *Cd4*‐Cre transgenic mice in C57BL/6 background from The Jackson Laboratory to produce age‐ and sex‐matched *Hectd1* ^+/+^ *Cd4*‐Cre and *Hectd1* ^fl/fl^ *Cd4*‐Cre mice for experiments. RAG‐1–deficient mice in C57BL/6 background were purchased from The Jackson Laboratory. All mice were housed in a specific pathogen‐free (SPF) environment with a 12‐12 light‐dark cycle, stable temperature (23 ± 2°C), and free access to food and water. All the mice were housed and used in adherence to protocols approved by the Institutional Animal Care and Use Committee (IACUC) of Shanghai Jiao Tong University School of Medicine. The Animal Use Protocol (AUP) Number is No. JUMC2023‐083‐A. The Laboratory Animal Production License Number is SCXK (Hu) 2023‐0010. The Laboratory Animal Use License Number is SYXK (Hu) 2023‐0041.

### 4.3 Plasmids, Antibodies, and Reagents

Plasmids have been described previously [57]. Flag‐tagged IGF2BP3 and HA‐tagged HECTD1 were cloned into the pLVX vector. His‐tagged WT, K48, K63 ubiquitin, and K63 mutant ubiquitin were cloned into the pcDNA3 vector. HIF‐1α was cloned into the MigR1 vector. Antibodies against HIF‐1α (36169s; 1:1000 for WB) and K63 polyubiquitin (D7A11; 1:1000 for WB) were purchased from Cell Signaling Technology. HRP‐conjugated anti‐HA antibody (3F10; 1:2000 for WB) was purchased from Roche. HRP‐conjugated anti‐Flag (M2, F3165; 1:2000 for WB) and anti‐β‐actin (A2228100UL; 1:10000 for WB) were purchased from MilliporeSigma. Anti‐His antibody (D291‐3; 1:1000 for WB) was obtained from Medical and Biological Laboratories. Anti‐HECTD1 antibody (A302‐908A; 1:1000 for WB) was purchased from Bethyl Laboratories. Anti‐YTHDC1 (Cat No. 14392‐1‐AP), Anti‐YTHDC2 (Cat No. 27779‐1‐AP), Anti‐YTHDF1 (Cat No. 17479‐1‐AP), and Anti‐YTHDF2 (Cat No. 24744‐1‐AP) were purchased from Proteintech. Anti‐YTHDF3 (A8395), Anti‐IGF2BP1 (A25715), Anti‐IGF2BP2 (A2749), Anti‐IGF2BP3 (A23295), Anti‐hnRNPA2B1 (A27189), Anti‐hnRNPC (A8958), and Anti‐hnRNPG (A21963) were purchased from ABclonal Technology. Anti‐m^6^A antibody was purchased from Synaptic Systems Inc. Anti‐eIF3A antibody (NBP1‐18891) was purchased from Novus Biologicals. For flow cytometric experiments, fluorochrome‐conjugated antibodies against CD4 (GK1.5), CD8 (53‐6.7), CD44 (IM7), CD62L (MEL‐14), CD69 (H1.2F3), Ki‐67 (So1A15), IFN‐γ (XMG1.2), IL‐17a (eBio17B7), and Foxp3 (FJK‐16s) were purchased from Thermo Fisher Scientific. Fluorochrome‐conjugated antibodies against IL‐10 (clone JES5‐16E3) and GM‐CSF (clone MP1‐22E9) were purchased from Biolegend. Anti‐mouse IL‐17A (clone 17F3) and isotype control IgG antibodies were purchased from Bio X Cell. RNase inhibitor (2313A) was purchased from Takara Inc.

### 4.4 Flow Cytometry

For cell surface markers staining, freshly isolated cells were stained with fluorochrome‐conjugated antibodies in PBS containing 2% FBS at 4°C for 30 min. For intracellular cytokine staining, cells were first stimulated with phorbol 12‐myristate 13‐acetate (PMA) and ionomycin in the presence of monensin for 5 h. Then, cells were stained with fluorochrome‐conjugated antibodies for surface markers and a fixable viability dye (eBioscience), next permeabilized with fixation/permeabilization buffer (Invitrogen) at 4°C for 1 h, followed by intracellular staining according to the manufacturer's protocol (Thermo Fisher Scientific). Samples were acquired on a BD LSRFortessa X‐20 flow cytometer (BD Biosciences), and flow cytometric data were analyzed using FlowJo software (version 10.8.1, BD Life Sciences).

### 4.5 RNA Sequencing Analysis

CD4^+^ T cells were isolated from 6‐8‐week‐old *Hectd1* ^+/+^ *Cd4*‐Cre and *Hectd1* ^fl/fl^ *Cd4*‐Cre mice and stimulated with 1 µg/mL α‐CD3 and 1 µg/mL α‐CD28 antibodies for 24 h. Total RNA was isolated with TRIzol (Invitrogen) and subjected to RNA sequencing using Illumina NovaSeq 6000 (150 bp paired‐end reads). Raw bulk RNA‐seq reads were aligned to the mouse reference genome (mm10) using HISAT2 (v2.0.5) with default parameters, with an average mapping rate of approximately 96% across all samples. The resulting alignment files were processed using HTSeq to generate gene‐level read counts. Raw read counts were normalized using the R package DESeq2 (v1.32.0), which was subsequently used for differential expression analysis. For visualization, normalized expression values were centered and scaled for each gene to generate Z‐scores. KEGG pathway enrichment analysis was performed by OmicsBean software. For analysis of public datasets GSE193677 and GTEx, normalized gene expression matrices were used. Gene Set Enrichment Analysis (GSEA) was performed using the GSEA software. pTh17 signatures were defined as the mean expressions of *CXCL3*, *IL22*, *IL3*, *CCL4*, *GZMB*, *IRAG2*, *CCL5*, *CASP1*, *CSF2*, *CCL3*, *TBX21*, *ICOS*, *IL7R*, *STAT4*, *LGALS3*, and *LAG3*, whereas npTh17 signatures were defined as the mean expressions of *IL6ST*, *IL1RN*, *IKZF3*, *MAF*, *AHR*, *IL9*, and *IL10*. Spearman correlation analysis (“cor.test” function in R) was applied to evaluate associations between *HECTD1* expression and pTh17 signatures, npTh17 signatures, or *HIF1A* expression. The results were ultimately visualized as scatter plots.

### 4.6 Targeted Metabolomics

Targeted metabolomics was performed using CD4^+^ T cells from *Hectd1* ^+/+^ *Cd4*‐Cre and *Hectd1* ^fl/fl^ *Cd4*‐Cre following anti‐CD3/CD28 stimulation. For each sample, 3×10^6^ cells were collected, with three independent biological replicates per group. Cellular metabolites were extracted with 1 mL pre‐chilled 80% methanol, sonicated in an ice bath for 20 min, and incubated at −20°C for at least 1 h. After centrifugation at 16 000 × g for 20 min at 4°C, the supernatants were vacuum‐dried and reconstituted in 100 µL 50% methanol. Metabolites were quantified by UHPLC‐MS/MS using a Shimadzu Nexera X2 LC‐30AD system coupled to a QTRAP 6500+ mass spectrometer (AB SCIEX).

### 4.7 CD4^+^ T Cell Differentiation In Vitro

Naïve CD4^+^ T cells (CD4^+^CD44^low^CD62L^high^) were isolated from spleen and lymph nodes of 4‐6‐week‐old mice using the Mouse CD4 Naïve T cell Enrichment Kit (Invitrogen). Then the cells were cultured in RPMI 1640 medium supplemented with 10% FBS and 1% penicillin plus streptomycin, 50 µM 2b‐mercaptoethanol, 2 mM L‐Glutamine (Gibco), 1x Nonessential amino acid (Gibco, 100x), 10 mM HEPES (Gibco), 1 mM sodium pyruvate (Gibco) and differentiated in 96‐well plates coated with α‐CD3/α‐CD28 antibodies in the presence of different cytokine cocktails: Th1: 20 ng/mL IL‐12 (R&D) and 5 µg/mL α‐IL‐4 (BioLegend); Th2: 20 ng/mL IL‐2 (R&D), 50 ng/mL IL‐4 (R&D) and 10 µg/mL α‐IFN‐γ (BioLegend); Th17: 1 ng/mL TGF‐β (R&D), 20 ng/mL IL‐6 (R&D), 25 ng/mL IL‐23 (R&D), 10 ng/mL IL‐1β (R&D), 5 µg/mL α‐IL‐4 (BioLegend) and 10 µg/mL α‐IFN‐γ (BioLegend); iTreg: 10 ng/mL IL‐2 (R&D) and 5 ng/mL TGF‐β (R&D) for 96 h.

### 4.8 Colitis and CAC Model

For the dextran sulphate sodium (DSS)‐induced colitis model, age‐ and sex‐matched *Hectd1* ^+/+^ *Cd4*‐Cre and *Hectd1* ^fl/fl^ *Cd4*‐Cre mice were subjected to 2.5% DSS in sterile drinking water for 7 days, followed by normal drinking water. Clinical scores, micro‐colon endoscopy, colon length, hematoxylin and eosin (HE) staining of colon, mesenteric lymph nodes (mLN), and lamina propria lymphocytes (LPLs) were measured in each mouse at the end of the experiment. For the DSS/azoxymethane (AOM)‐induced CAC model, age‐ and sex‐matched *Hectd1* ^+/+^ *Cd4*‐Cre and *Hectd1* ^fl/fl^ *Cd4*‐Cre mice were injected intraperitoneally with AOM (10 mg/kg body weight) followed by three cycles of 2.5% DSS exposure. Micro‐colon endoscopy, colon tumors, HE staining of colon tumor, mLN, and tumor‐infiltrating lymphocytes (TILs) were measured in each mouse at the end of the experiment. Anti‐mouse IL‐17A (100 µg/injection) or isotype control (100 µg/injection) antibodies were injected intraperitoneally every three days for a total of five injections beginning with the second and third DSS exposure cycles.

### 4.9 Adoptive T Cell Transfer Model

For the adoptive T cell transfer‐induced colitis model, naïve CD4^+^ T cells (CD4^+^CD44^low^CD62L^high^) were isolated from spleen and lymph nodes of age‐ and sex‐matched *Hectd1* ^+/+^ *Cd4*‐Cre and *Hectd1* ^fl/fl^ *Cd4*‐Cre mice using Mouse CD4 Naïve T cell Enrichment Kit (Invitrogen), and 5×10^5^ cells per mice were intravenously injected into 8‐week‐old *Rag1* ^−/−^ recipient mice. *Rag1* ^−/−^ recipient mice were weighed every week and sacrificed at four weeks after receiving the transfer. Micro‐colon enteroscopy, colon pathology, and LPLs were measured in each mouse at the end of the experiment. For the adoptive T cell transfer combined with DSS/AOM‐induced CAC model, 8‐week‐old *Rag1*‐/‐ mice were intraperitoneally injected with AOM (10 mg/kg body weight) one week before adoptive transfer of naïve CD4^+^ T cells (CD4^+^CD44^low^CD62L^high^, 1×10^6^ cells per mice) isolated from age‐ and sex‐matched *Hectd1* ^+/+^ *Cd4*‐Cre and *Hectd1* ^fl/fl^ *Cd4*‐Cre mice. Mice were then subjected to three cycles of 2% DSS administration and sacrificed on day 80. TILs were measured in each mouse at the end of the experiment.

### 4.10 CRISPR/Cas9‐Mediated Gene Editing

For each electroporation, 0.3 nmol sgRNA (GenScript, China) was incubated with 6 µg recombinant Cas9 protein (KACTUS Biosystems, China) at room temperature for 10 min to form Cas9–sgRNA ribonucleoprotein (RNP) complexes. Subsequently, 4×10^6^ isolated CD4^+^ T cells were resuspended in 20 µL of P3 Primary Cell Nucleofector Solution from the P3 Primary Cell 4D‐Nucleofector X Kit S (Lonza) and combined with the preassembled RNP complexes. Electroporation was performed using a 4D‐Nucleofector X Unit (Lonza) according to the manufacturer's instructions. After electrotransformation, the cells were stimulated with α‐CD3/α‐CD28 in vitro for 48 h to detect the knockout efficiency through Western blotting and proceed to the subsequent experiments. The following sgRNA sequences were used in the studies: sg*NC*, CGTTGCGAATCCAC‐CCTATT; sg*Igf2bp3*‐1, CAAGGACGCTAAGATCCCGG; sg*Igf2bp3*‐2, GGACGCTAAGATCCCGGTGG; sg*Igf2bp3*‐3, GGAACTACATGGGAAACCGA; sg*Hif1a*‐1, GGGCACCGATTCGCCATGGA; sg*Hif1a*‐2, CGATTCGCCATGGAGGGCGC; sg*Hif1a*‐3, GCTAACAGATGACGGCGACA; sg*Hectd1*‐1, ACTCAGGTAACTTAAGATAG; sg*Hectd1*‐2, TGGACACAGGTGTTGACCGA.

### 4.11 Retroviral Transduction

HIF‐1α overexpression plasmid was co‐transferred with the pCL‐Eco helper plasmid into platE cells using Tenfect DNA transfection reagent (TEYE Corporation, China). The supernatants containing viruses were mixed with α‐CD3/α‐CD28‐activated CD4^+^ T cells (MOI = 5:1) with polybrene (1 µg/ml), and then the mixtures were added to a 24‐well plate. After centrifugation at 30°C, 3000 rpm, with acceleration 2 and deceleration 0 for 90 min, cells were cultured at 37 °C under 5% CO_2_ for 24 h. Infected cells were then used for Seahorse assay or in vitro differentiation.

### 4.12 Glycolytic and Mitochondrial Respiration Rate Measurement

CD4^+^ T cells were isolated, knocked out *Igf2bp3* or *Hif1a* using CRISPR/Cas9, transduced with HIF‐1α, treated with HIF‐1α agonist ML228 or treated with HIF‐1α inhibitor PX‐478, and followed with α‐CD3/α‐CD28 antibodies for 48 h in vitro stimulation. 2×10^5^ cells per well were plated to the XF Cell Mito or the XF Glycolytic stress test, in the presence of injected compounds: 10 mM glucose, 2 µM oligomycin, 50 mM 2‐DG, 1 µM carbonyl cyanide 4‐(trifluoromethoxy) phenylhydrazone (FCCP), 0.5 µM rotenone/antimycin A (all products from Agilent Technologies). Extracellular acidification rate (ECAR) and oxygen consumption rate (OCR) for each well were measured by Seahorse XFe96 Extracellular Flux Analyzer (Agilent Technologies) according to the manufacturer's instructions.

### 4.13 Immunoprecipitation, Immunoblot, and Ubiquitination Assays

Stimulated CD4^+^ T cells were rinsed with ice‐cold PBS and lysed in RIPA buffer containing protease inhibitor (Sigma‐Aldrich) and 1 mM PMSF for 30 min at 4°C, followed by centrifugation to obtain supernatant. For immunoprecipitation (IP), the supernatants were immunoprecipitated overnight at 4°C with the appropriate antibody and then incubated with washed protein A/G‐agarose (Santa Cruz Biotechnology) for another 2 h at 4°C. After incubation, immunoprecipitated proteins were washed three times with lysis buffer for 10 min each. Samples were denatured by the addition of SDS loading buffer and boiled for 10 min at 100°C, resolved by 6%‐12.5% SDS‐PAGE, and analyzed by immunoblotting (IB) with the indicated antibodies. For ubiquitination assay, ubiquitinated IGF2BP3 from stimulated CD4^+^ T cells was detected by IB using K63‐linked ubiquitin antibody. For ubiquitination assay in HEK293T cells, Flag‐IGF2BP3, HA‐HECTD1, along with His‐ubiquitin expression vectors, were co‐transfected into HEK293T cells, the ubiquitinated IGF2BP3 was examined by α‐FLAG IP and α‐His IB.

### 4.14 Real‐Time Quantitative PCR (qRT‐PCR)

Total RNA was extracted using TRIzol (Invitrogen) and reverse transcribed into complementary DNA (cDNA) using HiScript II Q RT SuperMix for qPCR (Vazyme, Nanjing, China) according to the manufacturer's instructions. qPCR was performed on a 7500 Fast Real‐Time PCR System or ViiA7 Real‐Time PCR System using Hieff qPCR SYBR Green Master Mix (Yeasen Biotechnology, Shanghai, China) with gene‐specific primers. Data were generated through the comparative threshold cycle (ΔCt) method with normalization to the reference gene *b‐Actin*. Primers used for human were as follows: *ACTB* forward (F), CACCATTGGCAATGAGCGGTTC, *ACTB* reverse (R), AGGTCTTTGCGGATGTCCACGT; *HECTD1*‐F, GAGAAGGCACTGT‐CACAGGAGA, *HECTD1*‐R, GATGCCACTGTATCAGGGTCGT; *HIF1A*‐F, TATG‐AGCCAGAAGAACTTTTAGGC, *HIF1A*‐R, CACCTCTTTTGGCAAGCATCCTG; *IL17A*‐F, CGGACTGTGATGGTCAACCTGA, *IL17A*‐R, GCACTTTGCCTCCCAG‐ATCACA. Primers used for mouse were as follows: *Hectd1*‐F, TGACAATGTGGAT‐CGCTGTTT, *Hectd1*‐R, TACTATCCTCCGGGTGCATTC; *b‐Actin*‐F, CGTGAAAA‐GATGACCCAGATCA, *b‐Actin*‐R, CACAGCCTGGATGGCTACGT; *Tpi*‐F, CACC‐GCTTACATCGACTTTGC, *Tpi*‐R, GAAGGCCCCATTGGTCACTT; *Pgk1*‐F, ATGTCGCTTTCCAACAAGCTG, *Pgk1*‐R, GCTCCATTGTCCAAGCAGAAT; *Eno1*‐F, TGCGTCCACTGGCATCTAC, *Eno1*‐R, CAGAGCAGGCGCAATAGTTTT‐A; *Pkm*‐F, CGCCTGGACATTGACTCTG, *Pkm*‐R, GAAATTCAGCCGAGCCACA‐TT; *Gapdh*‐F, TGACCTCAACTACATGGTCTACA, *Gapdh*‐R, CTTCCCATTCTCG‐GCCTTG; *Gpi1*‐F, TGACTGGAAAGGGTACACTGG, *Gpi1*‐R, TGGGTCCCATC‐AATGTTAGAGAC; *Pfkl*‐F, GAACTACGCACACTTGACCAT, *Pfkl*‐R, CTCCAAA‐ACAAAGGTCCTCTGG; *Tfrc*‐F, GTTTCTGCCAGCCCCTTATTAT, *Tfrc*‐R, GCAA‐GGAAAGGATATGCAGCA; *Hif1a*‐F, TCTCGGCGAAGCAAAGAGTC, *Hif1a*‐R, AGCCATCTAGGGCTTTCAGATAA; *Rora*‐F, GTGGAGACAAATCGTCAGGAAT, *Rora*‐R, TGGTCCGATCAATCAAACAGTTC; *Rorc*‐F, TCCACTACGGGGTTATC‐ACCT, *Rorc*‐R, AGTAGGCCACATTACACTGCT; *Il17a*‐F, TCAGCGTGTCCAAA‐CACTGAG, *Il17a*‐R, CG‐CCAAGGGAGTTAAAGACTT; *Il17f*‐F, TGCTACTGTT‐GATGTTGGGAC, *Il17f*‐R, CAGAAATGCCCTGGTTTTGGT; *Il21*‐F, GGACCCTT‐GTCTGTCTGGTAG, *Il21*‐R, TGTGGAGCTGATAGAAGTTCAGG; *Il22*‐F, ATGAGTTTTTCCCTTATGGGGAC, *Il22*‐R, GCTGGAAGTTGGACACCTCAA.

### 4.15 Polyribosome Real‐Time qPCR

Isolated CD4^+^ T cells from age‐ and sex‐matched *Hectd1* ^+/+^ *Cd4*‐Cre and *Hectd1* ^fl/fl^ *Cd4*‐Cre mice were stimulated with α‐CD3/α‐CD28 antibodies for 24 h or differentiated Th17 cells and treated with 100 µg/mL cycloheximide (CHX) for 15 min. Then, 1×10^8^ CD4^+^ T cells per group were collected and lysed in polysome extraction buffer (20 mM Tris‐HCl, pH 7.5; 100 mM KCl; 5 mM MgCl_2_; 0.5% NP‐40; 100 µg/mL CHX) containing protease inhibitor and 200 U/mL RNase inhibitor. After lysis, 10% of the cell lysate was used as input RNA, the remainder was loaded into 5%–50% linear sucrose gradients which were prepared using a Gradient Master (BioComp) according to the manufacturer's instructions and separated by ultracentrifugation with a SW41 rotor (Beckman) at 270 000g at 4°C for 3 h. The distribution of ribosomes on the gradients was recorded, and the polyribosome fractions were collected with a BioComp Piston Gradient Fractionator equipped with a Bio‐Rad Econo UV Monitor (set at 260 nm). The input RNAs and polyribosome RNAs were then extracted by TRIzol reagent and were used for qPCR analysis.

### 4.16 RNA immunoprecipitation (RIP)

Stimulated CD4^+^ T cells or differentiated Th17 cells were rinsed with ice‐cold PBS and lysed in IP lysis buffer (25 mM Tris‐HCl, pH 7.4; 150 mM KCl; 5 mM EDTA; 0.5 mM DTT; 0.5% NP‐40) containing protease inhibitor and 200 U/mL RNase inhibitor for 30 min on ice, followed by centrifugation to harvest supernatant. The supernatants were incubated with indicated antibodies or IgG overnight at 4°C and then incubated with washed protein A/G‐beads (Bimake) for another 2 h at 4°C. After incubation, the immunoprecipitated complex was washed three times with IP lysis buffer for 10 min each. Co‐precipitated RNAs were extracted by TRIzol reagent, followed by qPCR analysis.

### 4.17 m^6^A‐RIP‐qPCR

Polyadenylated mRNA was purified from total RNA of stimulated CD4^+^ T cells or differentiated Th17 cells with the Dynabeads mRNA Purification Kit (Invitrogen) according to the manufacturer's instructions. After purification, 10% of the purified polyadenylated mRNA was used as the input set, and the remainder was incubated and rotated overnight at 4°C with α‐m^6^A antibody or IgG in IP wash buffer (10 mM Tris‐HCl, pH 7.4; 150 mM NaCl; 0.1% NP‐40) containing 200 U/mL RNase inhibitor. Cleaned protein A/G beads (Bimake) were then added and incubated at 4°C for another 2 h. At the end of incubation, the immunoprecipitated beads/m^6^A antibody/mRNA complexes were rinsed extensively three times for 10 min each with ice‐cold IP rinse buffer. Co‐precipitated RNA was eluted by TRIzol (Invitrogen) again, followed by qPCR analysis.

### 4.18 Cleavage Under Targets & Release Using Nuclease (CUT&RUN) assay

WT CD4^+^ T cells were isolated and stimulated with α‐CD3/α‐CD28 antibodies for 24 h in vitro or differentiated Th17 cells for CUT&RUN assays using CUT&RUN Assay kit (Cell Signaling Technology). 1×10^5^ CD4^+^ T cells per reaction were collected, washed, and rotated with Concanavalin A magnetic beads at 4°C overnight with 5 µg anti‐HIF‐1α (Cell Signaling Technology) or anti‐rabbit IgG mAb Isotype control (Merck Millipore) antibodies, respectively. Next, pAG‐MNase enzyme, as activated by CaCl_2_ was incubated with the reactants. Ultimately, DNA was purified using DNA Purification Buffers and Spin Columns (Cell Signaling Technology) and quantified by qPCR.

### 4.19 Dual Luciferase Assay

HIF‐1α overexpression plasmid in combination with pGL4.10 containing *Hectd1* promoter region and pRL‐TK plasmids, or IGF2BP3 overexpression plasmid in combination with pGL4.10 containing *Hif1a* WT or Mutation region and pRL‐TK plasmids were co‐transfected into HEK293T cells using Tenfect DNA transfection reagent (TEYE Corporation, China). Cells were harvested to detect luciferase activity using Dual‐Luciferase Reporter Assay System Kit (Promega) at 48 h after transfection.

### 4.20 Statistical Analysis

Statistical analysis was performed using GraphPad Prism software (version 9.3). Two‐tailed unpaired Student's *t* tests were performed to compare parameters among two groups. One‐way ANOVA statistical analyses with Tukey`s multiple comparisons test were performed to compare the four groups. Two‐way ANOVA with Geisser‐Greenhouse correction was performed for continuous outcomes across two groups. Data are presented as mean and standard error of the mean (SEM), and n is indicated in figure legends. P values less than 0.05 were considered statistically significant.

## Author Contributions

SX, QH, FW, and YZ designed and performed the experiments, analyzed the statistics, and prepared the figures; HF, KL, and QZ contributed to the performance of the experiments. TZ, YS, FL, FY, and XZ provided clinical samples from patients. XY, HH, and RZ supervised the work and wrote the manuscript.

## Ethics Approvals

All studies involving human specimens were conducted in accordance with the relevant ethical regulations and were approved by the Clinical Research Ethics Committee of Ruijin Hospital (approval nos. 2022–239 and 2026–49). Written informed consent was obtained from all participants prior to sample collection. All animal experiments were conducted in accordance with protocols approved by the Institutional Animal Care and Use Committee of Shanghai Jiao Tong University School of Medicine (AUP no. JUMC2023‐083‐A).

## Conflicts of Interest

The authors declare no conflicts of interest.

## Supporting information




**Supporting File**: advs78339‐sup‐0001‐SuppMat.docx.

## Data Availability

The data that support the findings of this study are available from the corresponding author upon reasonable request.

## References

[1] C. Le Berre , S. Honap , and L. Peyrin‐Biroulet , “Ulcerative Colitis,” Lancet 402 (2023): 571–584, 10.1016/s0140-6736(23)00966-2. 37573077

[2] M. Dolinger , J. Torres , and S. Vermeire , “Crohn's Disease,” The Lancet 403, no. 10432 (2024): 1177–1191, 10.1016/s0140-6736(23)02586-2. 38437854

[3] M. F. Neurath , “Strategies for Targeting Cytokines in Inflammatory Bowel Disease,” Nature Reviews Immunology 24, no. 8 (2024): 559–576, 10.1038/s41577-024-01008-6. 38486124

[4] K. Rajamäki , A. Taira , R. Katainen , et al., “Genetic and Epigenetic Characteristics of Inflammatory Bowel Disease–Associated Colorectal Cancer,” Gastroenterology 161, no. 2 (2021): 592–607, 10.1053/j.gastro.2021.04.042. 33930428

[5] M. S. Nadeem , V. Kumar , F. A. Al‐Abbasi , M. A. Kamal , and F. Anwar , “Risk of Colorectal Cancer in Inflammatory Bowel Diseases,” Seminars in Cancer Biology 64 (2020): 51–60, 10.1016/j.semcancer.2019.05.001. 31112753

[6] S. C. Shah and S. H. Itzkowitz , “Colorectal Cancer in Inflammatory Bowel Disease: Mechanisms and Management,” Gastroenterology 162, no. 3 (2022): 715–730.e3, 10.1053/j.gastro.2021.10.035. PMC9003896 34757143

[7] T. A. Ullman and S. H. Itzkowitz , “Intestinal Inflammation and Cancer,” Gastroenterology 140, no. 6 (2011): 1807–1816.e1, 10.1053/j.gastro.2011.01.057. 21530747

[8] V. Solitano , C. N. Bernstein , I. Dotan , et al., “Shaping the Future of Inflammatory Bowel Disease: A Global Research Agenda for Better Management and Public Health Response,” Nature Reviews Gastroenterology & Hepatology 22, no. 6 (2025): 438–452, 10.1038/s41575-025-01063-x. 40263433

[9] W. Sun , Z. Li , X. Zhang , Q. Luo , L. Wei , and C. Xiao , “Biomaterial‐Based Therapeutic Strategies for Inflammatory Bowel Disease,” Biomaterials 324 (2026): 123462, 10.1016/j.biomaterials.2025.123462. 40494022

[10] Q. H. Yang and C. N. Zhang , “Comparative Study on the Pathogenesis of Crohn's Disease and Ulcerative Colitis,” World Journal of Gastroenterology 31, no. 19 (2025): 106406, 10.3748/wjg.v31.i19.106406. PMC12146918 40497094

[11] M. Leppkes , C. Becker , and I. I. Ivanov , “RORγ‐Expressing Th17 Cells Induce Murine Chronic Intestinal Inflammation via Redundant Effects of IL‐17A and IL‐17F,” Gastroenterology 136, no. 1 (2009): 257–267, 10.1053/j.gastro.2008.10.018. 18992745

[12] S. Wu , K.‐J. Rhee , E. Albesiano , et al., “A Human Colonic Commensal Promotes Colon Tumorigenesis via Activation of T Helper Type 17 T Cell Responses,” Nature Medicine 15, no. 9 (2009): 1016–1022, 10.1038/nm.2015. PMC3034219 19701202

[13] W.‐J. Chae , T. F. Gibson , D. Zelterman , L. Hao , O. Henegariu , and A. L. M. Bothwell , “Ablation of IL‐17A Abrogates Progression of Spontaneous Intestinal Tumorigenesis,” Proceedings of the National Academy of Sciences 107, no. 12 (2010): 5540–5544, 10.1073/pnas.0912675107. PMC2851824 20212110

[14] S. Kirchberger , D. J. Royston , O. Boulard , et al., “Innate Lymphoid Cells Sustain Colon Cancer Through Production of Interleukin‐22 in a Mouse Model,” Journal of Experimental Medicine 210, no. 5 (2013): 917–931, 10.1084/jem.20122308. PMC3646494 23589566

[15] K. Wang , M. K. Kim , G. di Caro , et al., “Interleukin‐17 Receptor a Signaling in Transformed Enterocytes Promotes Early Colorectal Tumorigenesis,” Immunity 41, no. 6 (2014): 1052–1063, 10.1016/j.immuni.2014.11.009. PMC4272447 25526314

[16] P. W. F. Karmaus , X. Chen , S. A. Lim , et al., “Metabolic Heterogeneity Underlies Reciprocal Fates of TH17 Cell Stemness and Plasticity,” Nature 565, no. 7737 (2019): 101–105, 10.1038/s41586-018-0806-7. PMC6420879 30568299

[17] R. Qiu , X. Yu , L. Wang , et al., “Inhibition of Glycolysis in Pathogenic TH17 Cells Through Targeting a miR‐21–Peli1 –c‐Rel Pathway Prevents Autoimmunity,” The Journal of Immunology 204, no. 12 (2020): 3160–3170, 10.4049/jimmunol.2000060. 32414810

[18] K. Xu , N. Yin , M. Peng , et al., “Glycolytic ATP Fuels Phosphoinositide 3‐Kinase Signaling to Support Effector T Helper 17 Cell Responses,” Immunity 54, no. 5 (2021): 976–987.e7, 10.1016/j.immuni.2021.04.008. PMC8130647 33979589

[19] V. A. Gerriets , R. J. Kishton , A. G. Nichols , et al., “Metabolic Programming and PDHK1 Control CD4^+^ T Cell Subsets and Inflammation,” Journal of Clinical Investigation 125, no. 1 (2015): 194–207, 10.1172/jci76012. PMC4382238 25437876

[20] L. Z. Shi , R. Wang , G. Huang , et al., “HIF1α–Dependent Glycolytic Pathway Orchestrates a Metabolic Checkpoint for the Differentiation of TH17 and Treg Cells,” Journal of Experimental Medicine 208, no. 7 (2011): 1367–1376, 10.1084/jem.20110278. PMC3135370 21708926

[21] L. E. A. Damasceno , D. S. Prado , F. P. Veras , et al., “PKM2 Promotes Th17 Cell Differentiation and Autoimmune Inflammation by Fine‐Tuning STAT3 Activation,” Journal of Experimental Medicine 217, no. 10 (2020): e20190613, 10.1084/jem.20190613. PMC7537396 32697823

[22] T. Abimannan , V. Parthibane , S.‐H. Le , et al., “Sphingolipid Biosynthesis is Essential for Metabolic Rewiring During T H 17 Cell Differentiation,” Science Advances 10, no. 17 (2024): eadk1045, 10.1126/sciadv.adk1045. PMC11042737 38657065

[23] J. J. Cho , Z. Xu , U. Parthasarathy , et al., “Hectd3 Promotes Pathogenic Th17 Lineage Through Stat3 Activation and Malt1 Signaling in Neuroinflammation,” Nature Communications 10, no. 1 (2019): 701, 10.1038/s41467-019-08605-3. PMC6370850 30741923

[24] G. Zhou , W. Wu , L. Yu , et al., “Tripartite Motif‐Containing (TRIM) 21 Negatively Regulates Intestinal Mucosal Inflammation Through Inhibiting TH1/TH17 Cell Differentiation in Patients With Inflammatory Bowel Diseases,” Journal of Allergy and Clinical Immunology 142, no. 4 (2018): 1218–1228.e12, 10.1016/j.jaci.2017.09.038. 29113905

[25] A. Chitrakar , S. A. Budda , J. G. Henderson , R. C. Axtell , and L. A. Zenewicz , “E3 Ubiquitin Ligase Von Hippel–Lindau Protein Promotes Th17 Differentiation,” The Journal of Immunology 205, no. 4 (2020): 1009–1023, 10.4049/jimmunol.2000243. PMC8167928 32690659

[26] K. Chathuranga , P. Rathnapala , A. Weerawardhana , et al., “The E3 Ubiquitin Ligase MARCH2 Controls TNF‐α Mediated Inflammation by Autoubiquitination,” Cell Communication and Signaling 23, no. 1 (2025): 257, 10.1186/s12964-025-02260-6. PMC12126869 40450320

[27] J. He , Y. Song , G. Li , et al., “Fbxw7 increases CCL2/7 in CX3CR1^hi^ Macrophages to Promote Intestinal Inflammation,” Journal of Clinical Investigation 129, no. 9 (2019): 3877–3893, 10.1172/jci123374. PMC6715366 31246581

[28] Q. Yu , S. Zhang , K. Chao , et al., “E3 Ubiquitin Ligase RNF183 is a Novel Regulator in Inflammatory Bowel Disease,” Journal of Crohn's and Colitis 10, no. 6 (2016): 713–725, 10.1093/ecco-jcc/jjw023. 26818663

[29] M. Kathania , P. Khare , M. Zeng , et al., “Itch Inhibits IL‐17‐Mediated Colon Inflammation and Tumorigenesis by ROR‐γt Ubiquitination,” Nature Immunology 17, no. 8 (2016): 997–1004, 10.1038/ni.3488. 27322655

[30] Y.‐M. Kim , H.‐Y. Kim , H. T. Ha Thi , et al., “Pellino 3 Promotes the Colitis‐Associated Colorectal Cancer Through Suppression of IRF4 ‐Mediated Negative Regulation of TLR4 Signalling,” Molecular Oncology 17, no. 11 (2023): 2380–2395, 10.1002/1878-0261.13475. PMC10620127 37341064

[31] K. R. James , T. Gomes , R. Elmentaite , et al., “Distinct Microbial and Immune Niches of the Human Colon,” Nature Immunology 21, no. 3 (2020): 343–353, 10.1038/s41590-020-0602-z. PMC7212050 32066951

[32] The GTEx Consortium , “The GTEx Consortium Atlas of Genetic Regulatory Effects Across Human Tissues,” Science 369 6509, (2020): 1318–1330, 10.1126/science.aaz1776. PMC7737656 32913098

[33] J. T. Low , Y. Kim , M. Matsushita , and P. C. Ho , “Role of Oxygen Sensing and Hypoxia‐Inducible Factors in Orchestrating Innate Immune Responses,” Nature Immunology 26, no. 12 (2025): 2138–2147, 10.1038/s41590-025-02317-1. 41203908

[34] H. Huang , H. Weng , W. Sun , et al., “Recognition of RNA N6‐Methyladenosine by IGF2BP Proteins Enhances mRNA Stability and Translation,” Nature Cell Biology 20, no. 3 (2018): 285–295, 10.1038/s41556-018-0045-z. PMC5826585 29476152

[35] K. Lv , C. Gong , C. Antony , et al., “HectD1 Controls Hematopoietic Stem Cell Regeneration by Coordinating Ribosome Assembly and Protein Synthesis,” Cell Stem Cell 28, no. 7 (2021): 1275–1290.e9, 10.1016/j.stem.2021.02.008. PMC8254759 33711283

[36] Z.‐W. Wang , Y.‐H. Li , M.‐Y. Cai , et al., “IGF2BP3 Promotes Autophagy‐Mediated TNBC Metastasis via m6A‐Dependent, Cap‐Independent C‐Met Translation,” Cell Communication and Signaling 23, no. 1 (2025): 303, 10.1186/s12964-025-02316-7. PMC12211798 40598509

[37] A. Wang , H. Huang , J.‐H. Shi , et al., “USP47 Inhibits m6A‐Dependent C‐Myc Translation to Maintain Regulatory T Cell Metabolic and Functional Homeostasis,” Journal of Clinical Investigation 133, no. 23 (2023): e169365, 10.1172/jci169365. PMC10688989 37788092

[38] N. Zheng and N. Shabek , “Ubiquitin Ligases: Structure, Function, and Regulation,” Annual Review of Biochemistry 86, no. 1 (2017): 129–157, 10.1146/annurev-biochem-060815-014922. 28375744

[39] D. Kealy , R. Ellerington , S. Bansal , et al., “JAK2V617F Reprograms Hypoxia Inducible Factor‐1 to Induce a Non‐Canonical Hypoxia Regulon in Myeloproliferative Neoplasms,” Leukemia 40, no. 3 (2026): 609–621, 10.1038/s41375-025-02843-9. PMC12960217 41629621

[40] G. Zerafati‐Jahromi , E. Oxman , H. D. Hoang , et al., “Sequence Variants in HECTD1 Result in a Variable Neurodevelopmental Disorder,” The American Journal of Human Genetics 112, no. 3 (2025): 537–553, 10.1016/j.ajhg.2025.01.001. PMC11947180 39879987

[41] Q. Li , P. Wang , Y. Gong , et al., “α‐Klotho Prevents Diabetic Retinopathy by Reversing the Senescence of Macrophages,” Cell Communication and Signaling 22, no. 1 (2024): 449, 10.1186/s12964-024-01838-w. PMC11426092 39327553

[42] S. Liao , Q. Zheng , H. Shen , et al., “HECTD1‐Mediated Ubiquitination and Degradation of Rubicon Regulates Autophagy and Osteoarthritis Pathogenesis,” Arthritis & Rheumatology 75, no. 3 (2023): 387–400, 10.1002/art.42369. 36121967

[43] Y. Li , B. Huang , H. Yang , et al., “Latexin Deficiency in Mice Up‐Regulates Inflammation and Aggravates Colitis Through HECTD1/Rps3/NF‐κB Pathway,” Scientific Reports 10, no. 1 (2020): 9868, 10.1038/s41598-020-66789-x. PMC7299958 32555320

[44] Y. Liu , X. Hu , S. Zhou , et al., “Golgi Protein 73 Promotes Angiogenesis in Hepatocellular Carcinoma,” Research (Wash D C) 7 (2024): 0425, 10.34133/research.0425. PMC11251733 39022745

[45] S. Wang , C. Wu , J. Wang , et al., “Hsa_circ_0002301 Inhibits Ferroptosis in Gastric Cancer by Encoding the de Novo Protein HECTD1‐463aa,” Journal of Translational Medicine 23, no. 1 (2025): 250, 10.1186/s12967-025-06226-7. PMC11871676 40022084

[46] A. Schnell , D. R. Littman , and V. K. Kuchroo , “TH17 Cell Heterogeneity and its Role in Tissue Inflammation,” Nature Immunology 24, no. 1 (2023): 19–29, 10.1038/s41590-022-01387-9. PMC10795475 36596896

[47] E. V. Dang , J. Barbi , H.‐Y. Yang , et al., “Control of TH17/Treg Balance by Hypoxia‐Inducible Factor 1,” Cell 146, no. 5 (2011): 772–784, 10.1016/j.cell.2011.07.033. PMC3387678 21871655

[48] Z. Zhao , Y. Wang , Y. Gao , et al., “The PRAK‐NRF2 Axis Promotes the Differentiation of Th17 Cells by Mediating the Redox Homeostasis and Glycolysis,” Proceedings of the National Academy of Sciences 120, no. 19 (2023): e2212613120, 10.1073/pnas.2212613120. PMC10175746 37126714

[49] S. Ma , Y. Ming , J. Wu , and G. Cui , “Cellular Metabolism Regulates the Differentiation and Function of T‐Cell Subsets,” Cellular & Molecular Immunology 21, no. 5 (2024): 419–435, 10.1038/s41423-024-01148-8. PMC11061161 38565887

[50] F. J. Gonzalez , C. Xie , and C. Jiang , “The Role of Hypoxia‐Inducible Factors in Metabolic Diseases,” Nature Reviews Endocrinology 15, no. 1 (2018): 21–32, 10.1038/s41574-018-0096-z. PMC6624429 30275460

[51] C. Xu and W. Dong , “Role of Hypoxia‐Inducible Factor‐1α in Pathogenesis and Disease Evaluation of Ulcerative Colitis,” Experimental and Therapeutic Medicine 11, no. 4 (2016): 1330–1334, 10.3892/etm.2016.3030. PMC4812492 27073444

[52] S. Yu , B. Li , J. Hao , et al., “Correlation of Hypoxia‐Inducible Facto‐1α and C‐Reactive Protein With Disease Evaluation in Patients With Ulcerative Colitis,” American Journal of Translational Research 12 (2020): 7826–7835. PMC7791512 33437363

[53] J. Ben‐Shoshan , S. Maysel‐Auslender , A. Mor , G. Keren , and J. George , “Hypoxia Controls CD4^+^CD25^+^ Regulatory T‐Cell Homeostasis via Hypoxia‐Inducible Factor‐1α,” European Journal of Immunology 38, no. 9 (2008): 2412–2418, 10.1002/eji.200838318. 18792019

[54] E. T. Clambey , E. N. McNamee , J. A. Westrich , et al., “Hypoxia‐Inducible Factor‐1 Alpha–Dependent Induction of FoxP3 Drives Regulatory T‐Cell Abundance and Function During Inflammatory Hypoxia of the Mucosa,” Proceedings of the National Academy of Sciences 109, no. 41 (2012): E2784–2793, 10.1073/pnas.1202366109. PMC3478644 22988108

[55] J. H. Lee , C. Elly , Y. Park , and Y. C. Liu , “E3 Ubiquitin Ligase VHL Regulates Hypoxia‐Inducible Factor‐1α to Maintain Regulatory T Cell Stability and Suppressive Capacity,” Immunity 42, no. 6 (2015): 1062–1074, 10.1016/j.immuni.2015.05.016. PMC4498255 26084024

[56] S. E. Corcoran and L. A. O'Neill , “HIF1α and Metabolic Reprogramming in Inflammation,” Journal of Clinical Investigation 126, no. 10 (2016): 3699–3707, 10.1172/jci84431. PMC5096812 27571407

[57] F. Wang , Y. Zhang , X. Yu , et al., “ZFP91 Disturbs Metabolic Fitness and Antitumor Activity of Tumor‐Infiltrating T Cells,” Journal of Clinical Investigation 131, no. 19 (2021): e144318, 10.1172/jci144318. PMC8483753 34403361

