# Supplementary material for: HECTD1 Directs HIF‐1α to Facilitate Intestinal Inflammation and Carcinogenesis via Promoting Th17 Differentiation and Glycolysis
Source: Adv Sci (Weinh). 2026 Oct 18:e78339. Online ahead of print. doi: 10.1002/advs.78339 (PMC13677005; doi:10.1002/advs.78339)
Supplement: Supplementary file 1 [file ADVS-9999-e78339-s001.docx]

**Supplementary information**

**HECTD1 Directs HIF-1α to Facilitate Intestinal Inflammation and Carcinogenesis via Promoting Th17 Differentiation and Glycolysis**

Shuiyu Xu^1,6^, Qiaoqiao Han^2,6^, Feixiang Wang^2,6^, Yuerong Zhang^3,6^, Haoran Feng^1,6^, Kun Liu^1^, Tao Zhang^1^, Yiqing Shi^1^, Fang Xiu Luo^4^, Fei Yuan^4^, Xuesong Zhao^5^, Qiang Zou^2^, Xiaoyan Yu^2,7,*^, Haiyan Huang^1,7,*^, and Ren Zhao^1,7,*^

^1^Department of General Surgery, Ruijin Hospital, Shanghai Jiao Tong University School of Medicine, 197 Ruijin Er Road, Shanghai 200025, China;

^2^Shanghai Institute of Immunology, Department of Immunology and Microbiology, State Key Laboratory of Systems Medicine for Cancer, Shanghai Jiao Tong University School of Medicine, 280 South Chongqing Road, Shanghai 200025, China;

^3^Department of Laboratory Medicine, Institute of Laboratory Medicine, Sichuan Provincial People's Hospital, School of Medicine, University of Electronic Science and Technology of China, Chengdu, China;

^4^Department of Pathology, Ruijin Hospital, Shanghai Jiao Tong University School of Medicine, 197 Ruijin Er Road, Shanghai 200025, China;

^5^Department of Radiology, Ruijin Hospital, Shanghai Jiao Tong University School of Medicine, 197 Ruijin Er Road, Shanghai 200025, China

^6^These authors contributed equally to this work.

^7^Lead contact

^*^**Correspondence**: Ren Zhao, zhaorensurgeon@aliyun.com; Haiyan Huang, haiyan_huang1003@163.com; Xiaoyan Yu, yxypeony@163.com.

The authors have declared that no conflict of interest exists.

**Supplementary Figure Legends**

**Figure S1.** Construction of *Hectd1* T cell-conditional knockout mice. (A) Quantification of the immunoblotting results shown in Figure 1E. HECTD1 protein expression was normalized to β-actin in the indicated *in vitro* differentiated CD4⁺ T-cell subsets. Data were obtained from three independent experiments. (B) Schematic diagram of *Hectd1* gene targeting strategy using a LoxP targeting system, and breeding strategy of *Hectd1* T cell-conditional knockout (*Hectd1*^fl/fl^*Cd4*-Cre) mice. (C) IB analysis of HECTD1 in *Hectd1*^+/+^*Cd4*-Cre (WT) and *Hectd1*^fl/fl^*Cd4*-Cre (KO) CD4^+^ T cells stimulated with α-CD3/α-CD28 for 24h. (D-F) 8-week-old *Hectd1*^+/+^*Cd4*-Cre and *Hectd1*^fl/fl^*Cd4*-Cre mice were provided with drinking water containing 2.5% DSS for 7 days, followed by regular water. Disease score (D; n = 8), images of mini-endoscopic colon (E), and representative hematoxylin and eosin (HE) staining of colon (F) were analyzed. (G-I) 8-week-old *Hectd1*^+/+^*Cd4*-Cre and *Hectd1*^fl/fl^*Cd4*-Cre mice received a single intraperitoneal (i.p.) injection of AOM followed by three cycles of 2.5% DSS drinking water exposure. Mice were sacrificed at day 80. Representative images of mini-endoscopic colon (H), percentage of tumors of indicated size (G), and representative HE staining of colon (I) were analyzed. Data are representative of three independent experiments (C-I). *, P < 0.05; **, P < 0.01. one-way ANOVA with Tukey’s multiple comparisons (A), or Two-tailed Student’s *t* test (D).

**Figure S2.** Phenotype of CD4^+^ T cells in *Hectd1*^fl/fl^*Cd4*-Cre mice. (A) Summary graphs of flow cytometric analysis of the percentages of CD4^+^CD8^-^ (CD4), CD4^-^CD8^+^ (CD8), CD4^-^CD8^-^ (DN), and CD4^+^CD8^+^ (DP) T cells in the thymus and CD4 and CD8 T cells in the spleen or peripheral lymph nodes (pLN) of 6-week-old *Hectd1*^+/+^*Cd4*-Cre and *Hectd1*^fl/fl^*Cd4*-Cre mice (n = 5). (B) Summary graphs of flow cytometric analysis of the percentages of effector CD4^+^ (CD44^hi^CD62L^lo^) and CD8^+^ (CD44^hi^) T cells in the spleen or pLN of 6-week-old *Hectd1*^+/+^*Cd4*-Cre and *Hectd1*^fl/fl^*Cd4*-Cre mice (n = 5). (C) Summary graphs of flow cytometric analysis of the percentages of IFN-γ-producing and IL-17a-producing CD4^+^ T cells in the spleen or pLN of 6-week-old *Hectd1*^+/+^*Cd4*-Cre and *Hectd1*^fl/fl^*Cd4*-Cre mice (n = 6). (D-F) Summary graphs of flow cytometric analysis of the cell activation (D), proliferation (E), and apoptosis (F) of CD4^+^ T cells from *Hectd1*^+/+^*Cd4*-Cre and *Hectd1*^fl/fl^*Cd4*-Cre mice stimulated with α-CD3/α-CD28 (n = 5). (G) Summary graphs of flow cytometric analysis of the percentages of *in vitro* differentiated Th1 and Treg cells from 6-week-old *Hectd1*^+/+^*Cd4*-Cre and *Hectd1*^fl/fl^*Cd4*-Cre mice (n = 4 or 6). (H) Spearman’s correlation of mRNA expression of *HECTD1* and pathogenic Th17 signature genes (pTh17, left) and non-pathogenic Th17 signature genes (npTh17, right) in whole blood of healthy individuals (n = 803) from the Genotype-Tissue Expression (GTEx) database. (I) qRT-PCR analysis of mRNA levels of Th17 cell signature genes in differentiated Th17 cells from 6-week-old *Hectd1*^+/+^*Cd4*-Cre and *Hectd1*^fl/fl^*Cd4*-Cre mice (n = 4). (J) Summary graphs of flow cytometric analysis of the percentages of CD11b^+^F4/80^+^ cells in the lamina propria lymphocytes (LPLs) from *Hectd1*^+/+^*Cd4*-Cre and *Hectd1*^fl/fl^*Cd4*-Cre mice in DSS-induced acute colitis model (n = 8). (K) Summary graphs of flow cytometric analysis of the percentages of IFN-γ-producing and Foxp3-positive CD4^+^ T cells in LPLs from *Hectd1*^+/+^*Cd4*-Cre and *Hectd1*^fl/fl^*Cd4*-Cre mice in DSS-induced acute colitis model (n = 6 or 8). (L) Summary graphs of flow cytometric analysis of the percentages of GM-CSF^+^IL-17a^+^- producing (left), IFN-γ^+^IL-17a^+^-producing (middle), and IL-10^+^IL-17a^+^-producing (right) CD4^+^ T cells in LPLs from *Hectd1*^+/+^*Cd4*-Cre and *Hectd1*^fl/fl^*Cd4*-Cre mice in DSS-induced acute colitis model (n = 6). (M) Summary graphs of flow cytometric analysis of the percentages of CD11b^+^F4/80^+^ cells in tumor-infiltrating lymphocytes (TILs) from *Hectd1*^+/+^*Cd4*-Cre and *Hectd1*^fl/fl^*Cd4*-Cre mice in DSS/AOM-induced CAC model (n = 8). (N) Summary graphs of flow cytometric analysis of the percentages of IFN-γ-producing and Foxp3-positive CD4^+^ T cells in TILs from *Hectd1*^+/+^*Cd4*-Cre and *Hectd1*^fl/fl^*Cd4*-Cre mice in DSS/AOM-induced CAC model (n = 8). (O) Summary graphs of flow cytometric analysis of the percentages of GM-CSF^+^IL-17a^+^- producing (left), IFN-γ^+^IL-17a^+^-producing (middle), and IL-10^+^IL-17a^+^-producing (right) CD4^+^ T cells in TILs from *Hectd1*^+/+^*Cd4*-Cre and *Hectd1*^fl/fl^*Cd4*-Cre mice in DSS/AOM-induced CAC model (n = 8). Data are representative of three independent experiments and are presented as mean ± SEM. ns, not statistically significant; *, P < 0.05; **, P < 0.01. Two-tailed Student’s t test (A-G, I, J, and M-O), or One-way ANOVA with Tukey’s multiple comparisons (K and L).

Figure S3. Validation of the colitis, CAC, and transcriptional phenotypes using mice *Hectd1*^fl/fl^ as controls. (A) Representative flow cytometric gating strategy for the identification and analysis of Th17 cells in Figure 2. (B-E) 8-week-old *Hectd1*^fl/fl^ and *Hectd1*^fl/fl^*Cd4*-Cre mice were provided with regular water or drinking water containing 2.5% DSS for 7 days, followed by regular water. Mice body weight loss (B), disease score (C), colon length (D), and the percentages of IL-17a-producing CD4+ T cells (E) in the LPLs were analyzed (n = 5). (F-H) 8-week-old *Hectd1*^fl/fl^ and *Hectd1*^fl/fl^*Cd4*-Cre mice received a single intraperitoneal (i.p.) injection of AOM followed by three cycles of 2.5% DSS drinking water exposure. Mice were sacrificed at day 80. Representative photographs of colorectal tumors (F), macroscopic colorectal tumor number (G; n = 4 or 3), and the percentages of IL-17a-producing CD4+ T cells (H; n = 3 or 6) in the TILs were analyzed. (I) qRT-PCR validation of the glycolysis and HIF-1α-related genes identified by RNA-seq analysis in Figure 3A. The expression levels of the indicated genes were determined in *Hectd1*^fl/fl^ and *Hectd1*^fl/fl^*Cd4*-Cre CD4⁺ T cells. Data are presented as mean ± SEM. *, P < 0.05; **, P < 0.01. Two-way ANOVA with Geisser-Greenhouse correction (B), or Two-tailed Student’s t test (C-E and G-I).

**Figure S4.** Pharmacological targeting of HIF-1α neutralizes the effects of HECTD1 deficiency in acute colitis. (A) Quantification of the immunoblotting results shown in Figure 4A. HIF-1α protein expression was normalized to β-actin in *Hectd1*^+/+^*Cd4*-Cre (WT) and *Hectd1*^fl/fl^*Cd4*-Cre (KO) CD4^+^ T cells stimulated with α-CD3/α-CD28 for 0, 3, 6, and 24h. Data were obtained from three independent experiments. (B-E) 8-week-old *Hectd1*^+/+^*Cd4*-Cre and *Hectd1*^fl/fl^*Cd4*-Cre mice received the DSS treatment, followed by intraperitoneal (i.p.) injection with saline or 0.25 mg/kg ML228. Mice were sacrificed at day 10. Representative HE staining of colon (B). Summary graphs of flow cytometric analysis of the percentages of GM-CSF^+^IL-17a^+^- producing (C), IL-10^+^IL-17a^+^-producing (D), IFN-γ-producing and Foxp3-positive (E) CD4^+^ T cells in LPLs. (F-H) 8-week-old *Hectd1*^+/+^*Cd4*-Cre and *Hectd1*^fl/fl^*Cd4*-Cre mice received DSS treatment, followed by intraperitoneal (i.p.) injection with saline or 40 mg/kg PX-478. Mice were sacrificed at day 10. Representative images of mini-endoscopic colon (F). Summary graph of colon length (G; n = 3 or 4) and flow cytometric analysis of the percentages of IL-17a-producing (left), IFN-γ-producing (middle), and Foxp3-positive (right) CD4^+^ T cells in the LPLs (H; n = 3 or 4). Data are representative of two independent experiments and are presented as mean ± SEM. ns, not statistically significant; *, P < 0.05; **, P < 0.01. Two-tailed Student’s t test (A), or One-way ANOVA with Tukey’s multiple comparisons (C-E, G, and H).

**Figure S5.** HECTD1 interacts with IGF2BP3 to promote HIF-1α translation. (A) qRT-PCR analysis of *Hif1a* mRNA remaining levels in *Hectd1*^+/+^*Cd4*-Cre and *Hectd1*^fl/fl^*Cd4*-Cre CD4^+^ T cells stimulated with α-CD3/α-CD28 in the presence of 5 μg/mL actinomycin D (Act.D) for 0, 1, 2, 4, or 16h (n = 3). (B) IB analysis of the indicated proteins in CD4^+^ T cells stimulated with α-CD3/α-CD28 for 24 h following incubation with CHX (50 μg/mL) for the indicated durations. (C) Polyribosome analysis: representative traces of ribosome extract from differentiated *Hectd1*^+/+^*Cd4*-Cre and *Hectd1*^fl/fl^*Cd4*-Cre Th17 cells in the presence of 100 μg/mL CHX (left); qRT-PCR analysis of relative expression ratio of polyribosome mRNAs to input mRNAs after sucrose gradient fraction of polyribosomes (right; n = 6). (D) m^6^A-RIP-qRT-PCR analysis of m^6^A levels of *Hif1a* mRNA in naïve CD4^+^ T cells and differentiated *Hectd1*^+/+^*Cd4*-Cre and *Hectd1*^fl/fl^*Cd4*-Cre Th17 cells (n = 6). (E) IB and IP analysis of HECTD1 using lysates from CD4^+^ T cells stimulated with α-CD3/α-CD28 for 24 h. (F) RIP analysis of the interaction of IGF2BP3 with *Hif1a* mRNA in differentiated *Hectd1*^+/+^*Cd4*-Cre and *Hectd1*^fl/fl^*Cd4*-Cre Th17 cells (n = 6). (G) Quantification of the immunoblotting results corresponding to Figure 5F. The co-immunoprecipitated EIF3A signal was quantified relative to β-actin in the corresponding cell lysates from *Hectd1*^+/+^*Cd4*-Cre (WT) and *Hectd1*^fl/fl^*Cd4*-Cre (KO) CD4⁺ T cells before (0 h) and after (24 h) activation. Data were obtained from three independent experiments. (H) IB and IP analysis of smear of IGF2BP3 using lysates from *Hectd1*^+/+^*Cd4*-Cre (WT) and *Hectd1*^fl/fl^*Cd4*-Cre (KO) CD4^+^ T cells stimulated with α-CD3/α-CD28 for 24h. (I) RIP analysis of the interaction of eIF3A with *Hif1a* mRNA in differentiated *Hectd1*^+/+^*Cd4*-Cre and *Hectd1*^fl/fl^*Cd4*-Cre Th17 cells (n = 6). (J) Dual luciferase reporter assay of HEK293T cells after co-transfection with IGF2BP3 overexpression plasmid and luciferase reporter plasmid containing *Hif1a* WT and mutation region (n = 4). Data are representative of three independent experiments and are presented as mean ± SEM. ns, not statistically significant; *, P < 0.05; **, P < 0.01. Two-way ANOVA with Geisser-Greenhouse correction (A), Two-tailed Student’s t test (C, D, F, and I), One-way ANOVA with Tukey’s multiple comparisons (G and J).

**Figure S6.** HECTD1 catalytic activity is essential for IGF2BP3 K63-linked ubiquitination. (A) IB and IP analysis of IGF2BP3 K63-linked ubiquitination in *Hectd1*^fl/fl^*Cd4*-Cre CD4^+^ T cells transduced with HECTD1 WT, Mut (HECTD1 C2579G lacking the E3 ligase activity), or empty vector. (B) IB and IP analysis of IGF2BP3 K63-linked ubiquitination in CD4^+^ T cells after electro-transfer into sg*NC* or sg*Hectd1*. (C and D) RIP analysis of the interaction of IGF2BP3 with *Hif1a* mRNA in *Hectd1*^fl/fl^*Cd4*-Cre CD4^+^ T cells transduced with HECTD1 WT, Mut (HECTD1 C2579G lacking the E3 ligase activity), or empty vector (C; n = 6), or CD4^+^ T cells after electro-transfer into sg*NC* or sg*Hectd1* (D; n = 6). (E) Lysates from *Hectd1*^fl/fl^*Cd4*-Cre CD4^+^ T cells transduced with HECTD1 WT, Mut (HECTD1 C2579G lacking the E3 ligase activity), or empty vector were subjected to IP with α-IGF2BP3 antibody. (F) Lysates from CD4^+^ T cells after electro-transfer into sg*NC* or sg*Hectd1* were subjected to IP with α-IGF2BP3 antibody. (G and H) RIP analysis of the interaction of eIF3A with *Hif1a* mRNA in *Hectd1*^fl/fl^*Cd4*-Cre CD4^+^ T cells transduced with HECTD1 WT, Mut (HECTD1 C2579G lacking the E3 ligase activity), or empty vector (G; n = 6), or CD4^+^ T cells after electro-transfer into sg*NC* or sg*Hectd1* (H; n = 6). (I) IB and IP analysis of IGF2BP3 K63-linked ubiquitination in HEK293T cells co-transfected with the indicated vectors. Data are representative of three independent experiments and are presented as mean ± SEM. ns, not statistically significant; **, P < 0.01. One-way ANOVA with Tukey’s multiple comparisons (C and G), Two-tailed Student’s t test (D and H).

**Figure S7.** *Igf2bp3* or *Hif1a* knockout abrogated the alleviating effect of colitis caused by *Hectd1* deficiency in CD4^+^ T cells. (A) IB analysis of IGF2BP3 in CD4^+^ T cells stimulated with α-CD3/α-CD28 for 24 h after electro-transfer into sg*NC* or sg*Igf2bp3*. (B) IB analysis of HIF-1α and HECTD1 in CD4^+^ T cells stimulated with α-CD3/α-CD28 for 24 h after electro-transfer into sg*NC* or sg*Hif1a*. (C) Quantification of the immunoblotting results shown in Figure 6A. HIF-1α protein expression was normalized to β-actin in *Hectd1*^+/+^*Cd4*-Cre (WT) and *Hectd1*^fl/fl^*Cd4*-Cre (KO) CD4^+^ T cells stimulated with α-CD3/α-CD28 for 24 h after electro-transfer into sg*NC* or sg*Igf2bp3*. Data were obtained from three independent experiments. (D-F) Naïve CD4^+^ T cells (CD4^+^CD44^low^CD62L^high^) isolated from 6-week-old *Hectd1*^+/+^*Cd4*-Cre and *Hectd1*^fl/fl^*Cd4*-Cre mice were adoptively transferred into 8-week-old Rag1^-/-^ mice after electro-transfer into sg*NC*, sg*Igf2bp3*, or sg*Hif1a*. Mice were sacrificed at day 30. Representative images of mini-endoscopic colon in Rag1^-/-^ recipient mice at day 30 (D). Summary graphs of flow cytometric analysis of the percentages of IFN-γ-producing, IL-17a-producing, and Foxp3-positive CD4^+^ T cells in mLN (E; n = 5). Summary graphs of flow cytometric analysis of the percentages of IFN-γ-producing and Foxp3-positive CD4^+^ T cells in LPLs (F; n = 5). Data are representative of three independent experiments (A-B) and are presented as mean ± SEM. ns, not statistically significant; **, P < 0.01. Two-tailed Student’s t test (C), or One-way ANOVA with Tukey’s multiple comparisons (E and F).

**Figure S8.** The positive feedback loop of HIF-1α and HECTD1 regulates Th17 cells. (A) CUT&RUN-qRT-PCR analysis of the interaction of HIF-1α with *Hectd1* promoter region using differentiated Th17 cells (n = 6). (B) Quantification of the CUT&RUN results corresponding to Figure 7C. Relative enrichment of HIF-1α at the *Hectd1* promoter was determined in CD4⁺ T cells. IgG was used as a negative control. Data were obtained from three independent experiments. (C) PCR analysis of the interaction of HIF-1α with *Hectd1* promoter region using differentiated Th17 cells. (D) Quantification of the CUT&RUN results corresponding to Figure S8C. Relative enrichment of HIF-1α at the *Hectd1* promoter was determined in differentiated Th17 cells. IgG was used as a negative control. Data were obtained from three independent experiments. (E and F) Spearman’s correlation of mRNA expression of *HECTD1* and *HIF1A* in whole blood of healthy individuals (n = 803) from the GTEx database (E) or a GEO dataset for IBD (GSE193677, F). Data are representative of three independent experiments (A and C) and are presented as mean ± SEM. *, P < 0.05; **, P < 0.01. Two-tailed Student’s t test (A, B, and D).
